# Multi-objective hybrid split-ring resonator and electromagnetic bandgap structure-based fractal antennas using hybrid metaheuristic framework for wireless applications

**DOI:** 10.1038/s41598-024-53443-z

**Published:** 2024-02-08

**Authors:** SatheeshKumar Palanisamy, S Saranya Rubini, Osamah Ibrahim Khalaf, Habib Hamam

**Affiliations:** 1 Department of ECE, BMS Institute of Technology and Management, Bengaluru, 560064 India; 2 Department of Computer Science and Engineering, PES University, Bengaluru, India; 3 Department of Solar, Al-Nahrain Research Center for Renewable Energy, Al-Nahrain University, Jadriya, Baghdad, Iraq; 4 Faculty of Engineering, Uni de Moncton, Moncton, NB E1A 3E9 Canada; 5 International Institute of Technology and Management (IITG), Avenue des Grandes Ecoles, 1989, Libreville, Gabon; 6 Bridges for Academic Excellence, Tunis 1002, Centre Ville, Tunisia; 7 Department of Electrical and Electronic Engineering Science, School of Electrical Engineering, University of Johannesburg, 2006 Johannesburg, South Africa

**Keywords:** Electrical and electronic engineering, Engineering

## Abstract

Design closure and parameter optimisation are crucial in creating cutting-edge antennas. Antenna performance can be improved by fine-tuning preliminary designs created using theoretical considerations and rough dimension adjustment via supervised parameter sweeps. This paper introduces a frequency reconfigurable antenna design that can operate at 28/38 GHz frequencies to meet FCC and Ofcom standards for 5G applications and in the 18 GHz frequency band for K-band radar applications. A PIN diode is used in this design to configure multiple frequency bands. The antenna has a modified rectangular patch-like structure and two optimised plugins on either side. The study that is being presented focuses on maximising the parameters that are subject to optimisation, including length (Ls), width (Ws), strip line width (W_1_), and height (ht), where the antenna characteristic parameters such as directivity is tuned by a hybrid optimisation scheme called Elephant Clan Updated Grey Wolf Algorithm (ECU-GWA). Here, the performance of gain and directivity are optimally attained by considering parameters such as length, width, ground plane length, width, height, and feed offsets X and Y. The bandwidth of the proposed antenna at − 10 dB is 0.8 GHz, 1.94 GHz, and 7.92 GHz, respectively, at frequencies 18.5 GHz, 28.1 GHz, and 38.1 GHz. Also, according to the simulation results, in the 18 GHz, 28 GHz, and 38 GHz frequencies S11, the return loss is − 60.81 dB, − 56.31 dB, and − 14.19 dB, respectively. The proposed frequency reconfigurable antenna simulation results achieve gains of 4.41 dBi, 6.33 dBi, and 7.70 dBi at 18.5 GHz, 28.1 GHz, and 38.1 GHz, respectively. Also, a microstrip quarter-wave monopole antenna with an ellipsoidal-shaped complementary split-ring resonator-electromagnetic bandgap structure (ECSRR-EBG) structure has been designed based on a genetic algorithm having resonating at 2.9 GHz, 4.7 GHz, 6 GHz for WLAN applications. The gain of the suggested ECSRR metamaterial and EBG periodic structure, with and without the ECCSRR bow-tie antenna. This is done both in the lab and with numbers. The measured result shows that the ECSRR metamaterial boosts gain by 5.2 dBi at 5.9 GHz. At 5.57 GHz, the two-element MIMO antenna achieves its lowest ECC of 0.00081.

## Introduction

The ever-growing advancements of the MIMO are speedily pushing the theory of “pervasive intelligence” to an extraordinary level. Numerous heterogeneous communication services should be appropriately integrated into a single device to formulate the massive MIMO vision better. The next generation of communication systems is expected to offer ultra-fast 5G infrastructure with high data rates and large system capacity, using the adequate bandwidth of the MMW spectrum^1^. To develop this infrastructure, efficient antenna front ends are needed to achieve the K/Ka-band highlighted by various R&D platforms^2^. Wideband antennas have always been an option to increase the system’s capacity. However, when achieving a larger bandwidth, there is a decrease in antenna efficiency in some cases. In addition, in terms of efficient use of the frequency spectrum, reconfigurable antennas are more valuable than wideband antennas. Since frequency reconfigurable antennas do not have to work in all bands simultaneously like wideband antennas, spectrum is used more efficiently.

Changes in the antenna geometry are made using different technologies so that the reconfigurable antennas can provide resonance at the desired operating frequency. This is highly effective in increasing cognitive system data throughput to increase next-generation communication networks’ overall adequate bandwidth and network capacity^3^. There are different studies in the literature on switching methods for reconfigurable antennas. These studies used PIN diodes, MEMS, FET varactors, and optical switches in planar patch antennas^4^. Factors such as insertion loss, reliability, insulation, linearity, and cost are considered when choosing the appropriate switching method. FETs and PIN diodes are well-structured and commercially accessible. PIN diodes provide low insertion loss, high isolation at high frequencies, and high powers.

MIMO antennas have become promising in the next generation of communication systems networks after their success in 4G networks. MIMO antenna arrays are independent communication channels that provide simultaneous signal output and support the desired level of communication^5^. Frequency-reconfigurable antennas are integrated into MIMO systems, providing adaptability to signal output and frequency selection. This is extremely important for the efficient use of the spectrum.

Multiband antennas are better technological solutions to arrive at this objective while reducing the dimensions of the R.F. front-end. Every antenna typically operates in two or one frequency band, and different antennas are needed for different appliances^6^. Fractal antennae are used in several industries, including commercial, military, and telecommunication appliances. As a result, there are many different ways to simulate a patch antenna, but MFA is one of the more well-known techniques. Because of these two features, the fractal antenna is lighter and smaller and can function in various frequency ranges.

The power allocation and antenna selection approach is one of the leading causes of multiple input and output systems^7^. GWO is used for optimal power allocation and to determine the optimal sizing of fuel cells^8^. PSO solves economic dispatch problems^9^. Table 1 summarises the literature relevant to the proposed antenna.

**Table 1:** Examining the relevant conventional microstrip patch antenna.

Relevant work	Adopted model	Features	Challenges
^10^	Four SRRs loaded in the superstrate	Good directivity Broader radiation pattern	Reduced gain and interferences
^11^	Finite element method model	Efficiency is high Antenna gain is better	Impedance mismatching leads reduction in return loss
^12^	Cantor set the clover model	Return loss is reduced Antenna gain is high	Very complex
^13^	Modified inter-digital capacitor (MIDC) model	Return loss is reduced Gain is high	More attention must be paid to antenna system miniaturization
^15^	CSRR metamaterial cell—particle swarm optimization-ANN model	Stable radiation pattern Peak gain of 6.3 dB Peak radiation efficiency of 98.3%	Requires computation of different dimensions
^16^	BF-PSO model	Utilization of time is low High bandwidth	We need to focus on the cost function
^17^	Hexagonal patch SRR inspired metamaterial substrate	compact array structure optimal reflection coeffecient	feasible switching technique to be employed
^18^	Genetic algorithm	The computational complexity is reduced The accuracy is high	The flexibility is not fixed at all
^19^	SRPA	The performance efficiency is enhanced High gain The size is miniaturized	It is used only for short ranges

The following is a list of the most critical findings from this research:

- Presents a revolutionary MFA design in which antenna characteristics like gain and directivity are carefully selected.
- Proposes a new Elephant Clan Updated Grey Wolf Algorithm (ECU-GWA) for performing optimization.

Table 1 reviews the conventional MFA designs. Initially, the SVR scheme was offered by Ref.^20^, which provided optimal impedance matching and higher antenna gain.

Nevertheless, it wants to contemplate SBD features with varied antenna geometries. Reference^21^ constructed a FEM model with good efficiency and gain. However, antenna matching was not considered. Cantor set technology^22^ optimal reflection coefficient and increased antenna directivity, but it is not easy. The Modified Square Sierpinski Gasket fractal antenna model^23^ improved benefits with low return loss. Antenna model miniaturization should be prioritized. The PSO-ANN model^24^ improves design accuracy and size minimization. Multidimensional calculations are required. The BF-PSO model^25^ ensures minimal time utilization and high bandwidth. However, consider the cost function. Although resonant frequency fluctuation was not detected, IWO improved reflection coeffeceint^26^. The FEM approach^27^ reduces array size and return loss. However, different switching technique should be used to choose frequencies. The GA technique^28^ has low processing complexity and high accuracy. The system’s fundamental issue is reduced flexibility. The SRPA’s efficiency and gain have improved since its deployment^29^, although it is only used at close ranges.

This document is structured in the following manner: “18 GHz and 28/38 GHz frequency reconfigurable antenna” section provides six frequency reconfigurable 5G and K-Band radar antenna designs and their optimization. “18 GHz and 28/38 GHz frequency reconfigurable antenna geometry” section describes design geometry and numerical and experimental evaluations of reconfigurable 5G and K-Band radar antennae. “Design of ECSRR bow-tie antenna” section demonstrates the design of the ECSRR bow-tie antenna, and “Reduction of mutual coupling using ECSRR EBG structure” section discusses the mutual coupling reduction technique using the EBG structure, concluding in “Conclusion” section.

## 18 GHz and 28/38 GHz frequency reconfigurable antenna

### Formulation of synthesizing issue

Assume a generic structure with an agitated fractal metal layer mounted upon a dielectric substrate of thickness, g typified by loss tangent $\delta$ and dielectric permittivity $\varepsilon_{r}$ with a metallic ground plane at the back. By portraying this geometry via feature vector h = {h_r_, r = 1, 2, 3…R} with every R (real-value) designing constraints or issue DoF (i.e., height, substrate, feed shape, width, and fractal dimension), the antenna synthesizing crisis could be fixed as below.

Multiband antenna synthesizing crisis^30^: Assign the value of the unidentified entry of h in the user-determined DoF limits in such a way that S_11_ (F;h) ≤ S_11_th for every F $\in$ {Fm,m = 1,2,3….M}, S_11_ (F;h) is the antenna dispersal constraint at a frequency F and S_11_th the related user determined parameter/prerequisite. In addition, M points out a band of interest count and Fmpoints to the central frequency of the mth (m = 1,2, …, M) band.

#### PRE block

**Figure 1:**
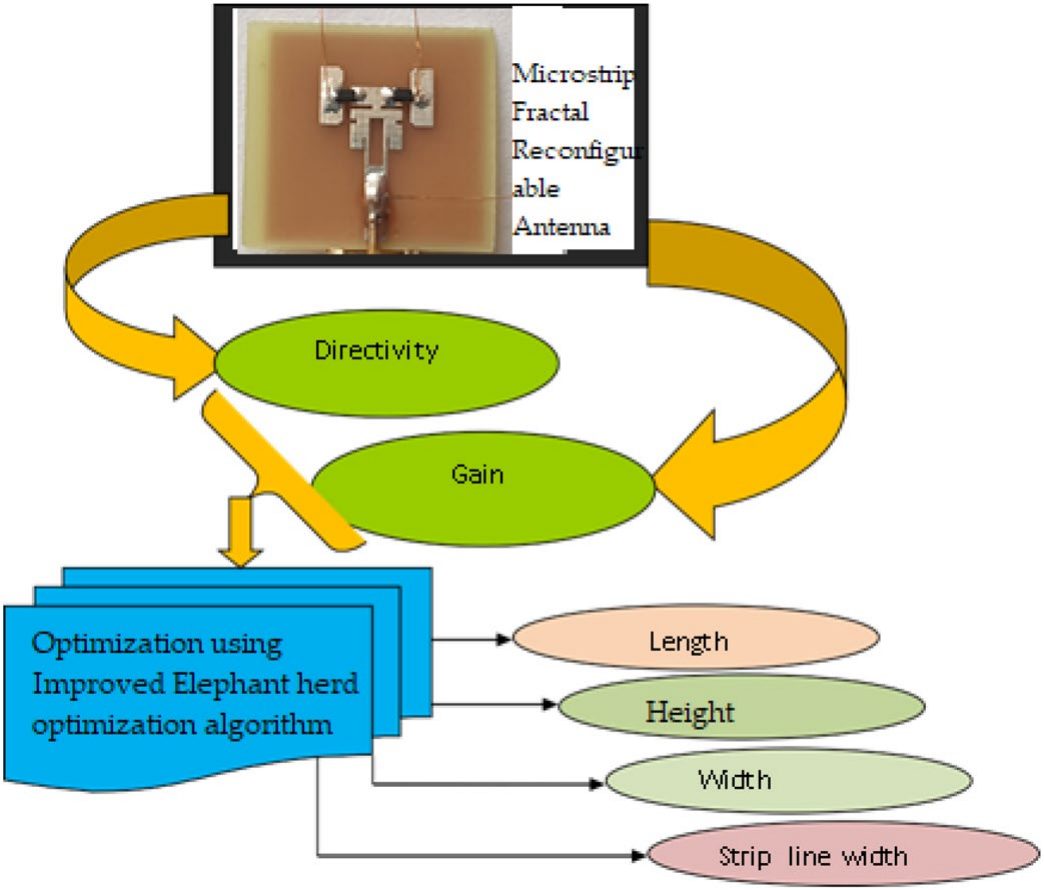
PRE-block implementation for microstrip patch antenna.

A model extracted from SVR^31^ is considered for the implementation of the PRE block, as shown in Fig. 1. After the offline training phase is done for learning the output-input relationships of the scheme to be followed beginning from a group of *T* training set couples [h^(t)^, $\vec{F}_{m} (h^{(t)} )$], a speedy online testing stage is further performed for emulating the physical method itself. Thus, the relationship between the approximated resonant frequency, $\hat{F}_{m} (h),m = 1,2,...M,$ and the antenna constraints *h* is designed as shown in Eq. (1), wherein, $\kappa (.)$ refers to a kernel function, $\alpha_{m} \underline{\underline{\Delta }} \left\{ {\alpha_{m}^{(t)} ,t = 1,2,...T} \right\}$ and $\beta_{m} \underline{\underline{\Delta }} \left\{ {\beta_{m}^{(t)} ,t = 1,2,...T} \right\}$ m = 1,2,..M refers to SVR weights and $\alpha_{m}$ *m*th frequency offset^32^. Figure 1 shows the overall depiction of the developed MFA design**.**

#### Offline SVR phase

For defining the training set (i.e., $\left[ {h^{(t)} ,\hat{F}_{m} \left( {h^{(t)} } \right)} \right];$ t = 1, 2….T, m = 1, 2…M), an O.A. scheme^33^ is deployed for determining the sample points, h^(t)^, t = 1, 2…T, m = 1, 2…M, of the functional space domain, $\left\{ {\hat{F}_{m} (h);m = 1,2...M} \right\}$ to be approximated via LBE. Indeed, it is renowned that such a technique permits sampling the space $\nu$ statistically constantly while reducing the dimension *T* of the training set. Comprehensively, the input training sample (t = 1, 2…T) is shown in Eqs. (2) and (3).

$$h^{(t)} = \left\{ {h_{r}^{(t)} ,r = 1,2,...R} \right\},$$

1 $$h_{r}^{(t)} = h_{r}^{\min } + \frac{{\varpi_{tr} }}{L - 1}\left( {h_{r}^{\max } - h_{r}^{\min } } \right),r = 1,2...R.$$

And $\varpi_{tr}$ points out the t,r component of a (L, T, R)-O.A.

$$\Omega \, = \;\left\{ {\varpi_{tr} \in N \cap \left[ {0,L - 1} \right];t = 1,...T;r = 1,2...R} \right\}.$$

The entry of Ω is built; therefore, they can presume a numeral value among [0, *L* − 1], *L* points out the quantization steps count selected for discretizing every constraint *h*_*r*_, *r* = 1, 2,… *R* in $\nu$.

The training stage is terminated by portraying the optimum values of $\varepsilon$-SVR constraint^34^

$${\text{s }}({\text{i}}.{\text{e}}.,\;\tilde{\alpha }_{m} \underline{\underline{\Delta }} \left\{ {\tilde{\alpha }_{m}^{(t)} ,t = 1,2...T} \right\},\tilde{\beta }_{m} \underline{\underline{\Delta }} \left\{ {\tilde{\beta }_{m}^{(t)} ,t = 1,2...T} \right\},\;\tilde{a}_{m} ;m = 1,2...M).$$

Consequently, the minimizing issue in Eq.  (5) is resolved using a local minimization scheme, *C* points out the kernel matrix, $\varepsilon$ and *Q* points out user-determined controlling constraints,

$$\left( {\tilde{\alpha }_{m} ,\tilde{\beta }_{m} } \right) = \mathop {\min }\limits_{{(\alpha_{m} ,\beta_{m} )}} \left[ {\frac{{\left( {\alpha_{m} - \beta_{m} } \right)^{\prime} C(\alpha_{m} - \beta_{m} )}}{2} + \varepsilon \sum\limits_{t = 1}^{T} {\left[ {\left( {\alpha_{m}^{(t)} + \beta_{m}^{(t)} } \right) + \sum\limits_{t = 1}^{T} {\tilde{F}_{m} \left( {h^{(t)} } \right)} \left( {\beta_{m}^{(t)} + \alpha_{m}^{(t)} } \right)} \right]} } \right].$$

2 $$\begin{aligned} {\text{ST }} & {\text{q1}}:\sum\limits_{t = 1}^{T} {\left( {\alpha_{m}^{(t)} - \beta_{m}^{(t)} } \right)} = 0 \\ & {\text{q2}}:0 \le \alpha_{m}^{(t)} \le Q;t = 1,2...T \\ & {\text{q3}}:0 \le \beta_{m}^{(t)} \le Q;t = 1,2...T. \\ \end{aligned}$$

### Elephant Clan Updated Grey Wolf Algorithm (ECU-GWA)

The study that is being presented focuses on maximizing the parameters that are subject to optimization, including length (*L*_*s*_), width (*W*_*s*_), strip line width (W_1_), and height (*ht*). The length is measured in millimeters. The devised method’s lower and upper bounds of length mm are 30 and 40, respectively. The bounds for width are 5 and 10, respectively, while the lower and higher bounds for the parameter height are set at 0.05 and 5, respectively. By optimizing these settings, the gain and directivity should increase. Equation  (6) illustrates the developed work’s goal, in which *G* depicts gain, and *Di* depicts directivity. The pseudocode for representing Elephant Clan Updated Grey Wolf Algorithm (ECU-GWA) is shown in Table 2.

$$Ob = Max\left[ {G,Di} \right].$$

**Table 2:**
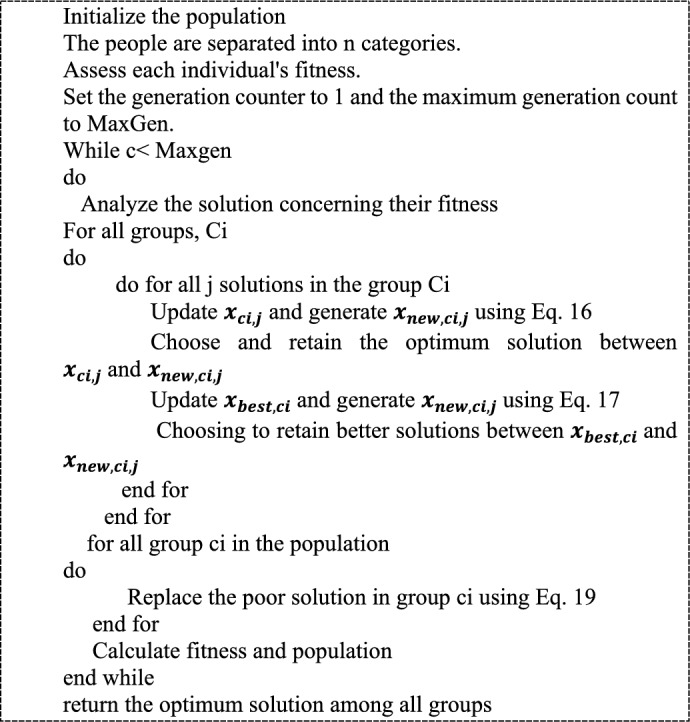
Algorithm 1 ECU-GWA algorithm.

The following lists the processes in the ECU-GWA:

3 $${\mathrm{ x}}_{{\text{new}},{\text{ci}},{\text{j}}}={{\text{x}}}_{{\text{ci}},{\text{j}}}+\mathrm{\alpha }\times \left({{\text{x}}}_{{\text{best}},{\text{ci}}}-{{\text{x}}}_{{\text{ci}},{\text{j}}}\right)\times {\text{r}},$$

$${{\text{x}}}_{{\text{new}},{\text{ci}},{\text{j}}}=\upbeta \times {{\text{x}}}_{{\text{center}},{\text{ci}}},$$

$${{\text{x}}}_{{\text{center}},{\text{ci}},{\text{d}}}=\frac{1}{{{\text{n}}}_{{\text{ci}}}}\times \sum_{{\text{j}}=1}^{{\text{d}}}{{\text{x}}}_{{\text{ci}},{\text{j}},{\text{d}}},$$

4 $${{\text{x}}}_{{\text{worst}},{\text{ci}}}={{\text{x}}}_{{\text{min}}}+\left({{\text{x}}}_{{\text{max}}}-{{\text{x}}}_{{\text{min}}}+1\right)\times {\text{rand}},$$

where x_ (new,c_i,j_) signifies the updated function of solution j within clan ci, is denoted as x_ (c_i_) denotes individual j’s prior position within clan ci.

To build an optimization problem, the degrees of freedom in the design must be precisely determined and parameterized. Furthermore, the design’s aims and restrictions must be quantified using a predefined set of parameters. Since no point in the Pareto set has both objectives better than any other, trade-offs between them also become optimal in this approach. For example, the set of parameters where the directivity is largest is not always where the standing-wave ratio is minimum.

Determining the parameters for cross-polarization, impedance matching, directivity, and frequency range is frequently a part of antenna design. It is possible to represent these parameters as constraint functions or objective functions. The optimisation problem’s classification is based on the characteristics of these functions. When designing antennas, the computer model used to tackle optimisation problems occasionally necessitates costly assessments. These analyses, which are sometimes referred to as Oracle queries, use numerical methods to estimate Maxwell’s equation^35^ answers. As a result, the optimisation pace and quantity are directly related.

As a result, the number of Oracle queries and the optimisation pace are strongly related. It is crucial to remember that optimisation is merely a tool and that the designer’s experience and intuition are still essential. Competent designers are able to identify important optimisation problems, reduce the number of viable solutions, and provide useful starting points. While narrowing the search space expedites optimisation, it may also cause issues with global optimality guarantees of the solution. The following examples of the Elephant Clan Updated Grey Wolf Algorithm (ECU-GWA) highlight and demonstrate each of these features.

### C.H. selection using adaptive sailfish optimizer

The Sailfish (S.F) matrix^36^ below lists the location of every single sailfish. They are designated as such with the initials injured S and Xielite SF. Those roles can affect how SFO operates and shorten the time needed to discover answers.

5 $${\text{SF}}_{{{\text{position}}}} = \left[ {\begin{array}{*{20}c} {{\text{SF}}_{1,1} } & {{\text{SF}}_{1,2} } & \cdots & {{\text{SF}}_{{1,{\text{n}}}} } \\ {{\text{SF}}_{2,1} } & {{\text{SF}}_{2,2} } & \cdots & {{\text{SF}}_{{2,{\text{n}}}} } \\ \vdots & \vdots & \vdots & \vdots \\ {{\text{SF}}_{{{\text{d}},1}} } & {{\text{SF}}_{{{\text{d}},2}} } & \cdots & {{\text{SF}}_{{d,{\text{n}}}} } \\ \end{array} } \right].$$

## 18 GHz and 28/38 GHz frequency reconfigurable antenna geometry

This section introduces a frequency reconfigurable antenna design that can operate at 28/38 GHz frequencies to meet FCC and Ofcom standards for 5G applications and in the 18 GHz frequency band for K-Band radar applications. A PIN diode is used in this design to configure multiple frequency bands. The antenna has a modified rectangular patch-like structure and two optimized plugins on either side. The attachment on both sides is switched with a highly insulated PIN diode. In cases where the plugins are separated from the antenna structure, the antenna structure operating in the 38 GHz and 28 GHz bands and the 18 GHz band also works if the plugins are included.

The bias voltage is applied to the diode terminals to operate the PIN diodes. The PIN diode prevents R.F. current from passing through in the “OFF” state; ideally, the S_21_parameter indicates high insulation. S_11_ parameter is expected to be 0 dB. On the other hand, the PIN diode is expected to work like a short circuit in the “ON” state, allowing maximum current to pass through. In the ideal case, the S_11_ parameter should be low for a better impedance match, and for 100% power delivery, the S_21_ parameter should be 0 dB. However, due to the losses in practical use, complete power transfer or reflection cannot be achieved.

The following mathematical formulas are used to determine the frequency at which the rectangular patch antenna will resonate^37^,

6 $$W_{p} = \frac{c}{{2f_{c} \sqrt {\frac{{\varepsilon_{r} + 1}}{2}} }},$$

7 $$L_{P} = \frac{c}{{2f_{0} \sqrt {\varepsilon_{reff} } }} - 2\Delta L,$$

8 $$\varepsilon_{reff} = \frac{{\varepsilon_{r} + 1}}{2} + \frac{{\varepsilon_{r} - 1}}{2}\left( {\frac{1}{{\sqrt {1 + 12\left( {\frac{H}{{W_{s} }}} \right)} }}} \right),$$

where Ws and H denote the substrate’s width and height, and ε_reff_ represents its effective permittivity.

When modelling, the following transmission line characteristic equations are taken into consideration for impedance matching^38^.

For $$\begin{gathered} \frac{{W_{f} }}{H} \le 1, \hfill \\ \hfill \\ \end{gathered}$$

9 $$Z_{0} = \frac{60}{{\varepsilon_{reff} }}\ln \left[ {\frac{8H}{{W_{f} }} + \frac{{W_{f} }}{4H}} \right],$$

where

$$\varepsilon_{reff} = \frac{{\varepsilon_{r} + 1}}{2} + \frac{{\varepsilon_{r} - 1}}{2}\left( {\frac{1}{{\sqrt {1 + 12\left( {\frac{H}{{W_{s} }}} \right)} }} + 0.04\left( {1 - \frac{{W_{f} }}{H}} \right)^{2} } \right).$$

For $$\frac{{W_{f} }}{H} \ge 1,$$

10 $$Z_{0} = \frac{{120\pi \sqrt {\varepsilon_{reff} } }}{{\frac{{w_{f} }}{H} + 1.393 + 0.667\ln \left( {\frac{{w_{f} }}{H} + 1.444} \right)}}.$$

Wf is the feed line’s width, and Z_0_ is the transmission line’s characteristic impedance. Equations  (11)– (13) are used to calculate the width (W_f_) and length (L_f_) of the feed network.

11 $$W_{f} = -\frac{2h}{\pi }\left( {ln (2B - 1)-B + 1} \right) + \frac{{\varepsilon_{r} - 1}}{{2\varepsilon_{r} }}\left[ {0.39+\ln \left( {B - 1} \right) - \frac{0.61}{{\varepsilon_{r} }}} \right],$$

12 $$L_{f} = \frac{\lambda }{{4\sqrt {\varepsilon_{reff} } }},$$

13 $$B = \frac{{60\pi^{2} }}{{Z_{0} \sqrt {\varepsilon_{r} } }}.$$

**Figure 2:**
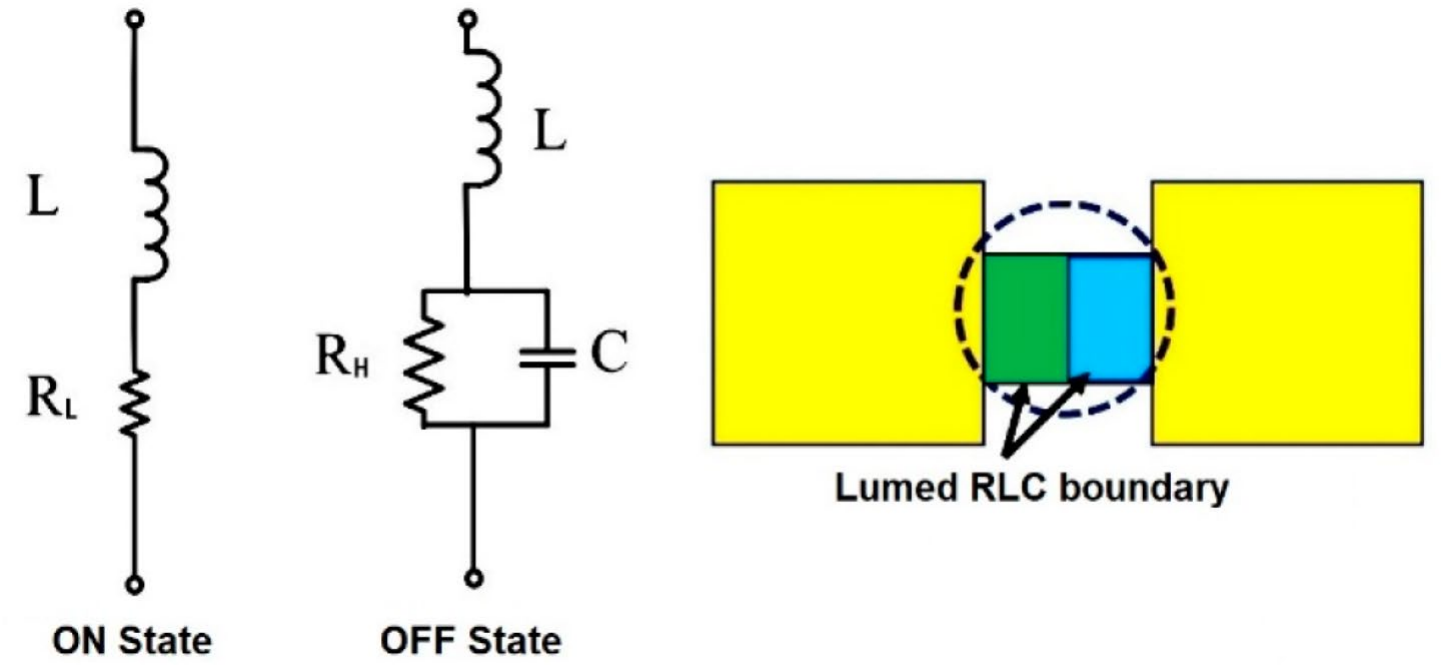
Equivalent circuits of P-i-N diode and its ANSYS model.

The proposed geometry is 35 µm with copper and 0.8 mm substrate thickness, double-sided FR-4 (ε_r_ = 4.3) material. With a length of 3.20 mm and a width of 1.46 mm, the supply line is designed to accommodate a 50 Ω impedance. Figure 2 shows the antenna geometry and placement patterns of the PIN diodes.

The primary radiator and 4 parasitic patches are inter-connected using pin diodes S_1_S_4_ in the radiating structure at the center. Pin diodes (Model SMP1345-079LF), as switches, enable/disable primary radiator/patch connections. Changing its effective resonant length connects or disconnects patches and the antenna—1 mm-width split parasitic patches of varied  dimensions on either side of the central radiator. Eight-pin-diode shifts between parasitic parts reconfigure patterns. These pin diodes vary in parasitic element length, which affects their operations (reflector, director, or both). These parasitic elements offer beam steering, adequate gain, and impedance matching for all operational modes (C). The suggested antenna’s parameters are shown in Table 3. The table below lists the antenna parameters, including length, width, strip line width, and feed parameters.

**Table 3:** Antenna geometry parameters.

Parameters	Dimensional values
Length of the patch (m)	0.0081
Width of the patch (m)	0.0055
Width of the strip line (m)	0.0034
Feed offset	[− 0.0240, − 0.0020]
Num iterations	2
Height (m)	0.0054
length of the groundplane (m)	0.0480
width of the groundplane (m)	0.0480
Feed location [x,y,z]	[− 0.0240, − 0.0020, 0.0054]
Fractal center offset [x,y]	[0, 0]
length of the substrate(m)	0.0480
width of the substrate(m)	0.0480
Substrate shape (m)	Box

### Switching technique

Pin diodes (SMP1345-079LF) act as potentiometers to alter resistance for any frequency band^39^. The resonanting dimensions are changed to reconfigure pattern and frequency. Figure 2 displays pin diode comparable circuits while ON and OFF. An ON-state RL series circuit comprises a meager resistance and an inductance, L. The OFF state of RLC is made up of an inductor L and a capacitor C. Skyworks SMP1345-079LF, a cheap and readily available p-i-n diode, is used for this research work. The calculated elemental values are L = 0.69 nH, C = 0.148 pF, and R_.L._ = 1.5 Ω.

**Figure 3:**
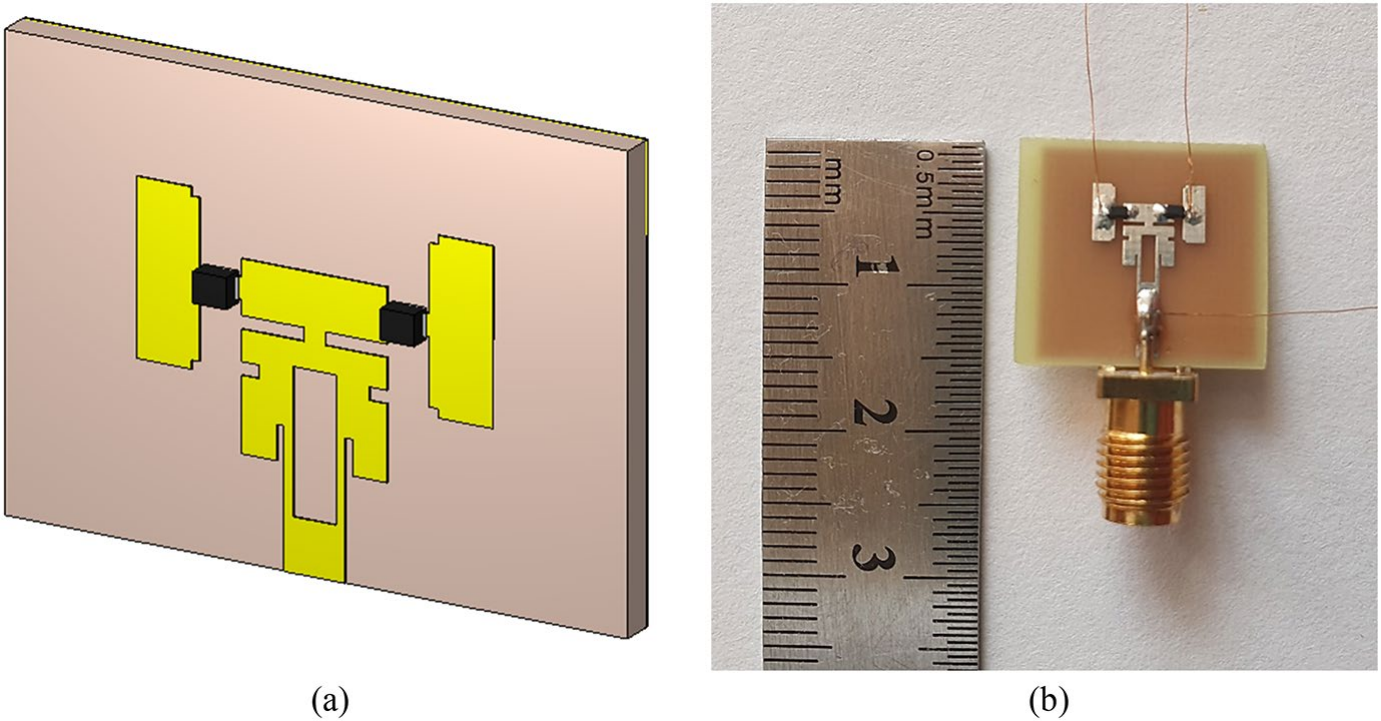
18 GHz and 28/38 GHz reconfigurable frequency antenna: (**a**) Simulated antenna geometry. (**b**) Prototype of the fabricated antenna.

The biasing circuit on the antenna’s rear operates the pin diode during measurement, as shown in Fig. 3.

**Figure 4:**
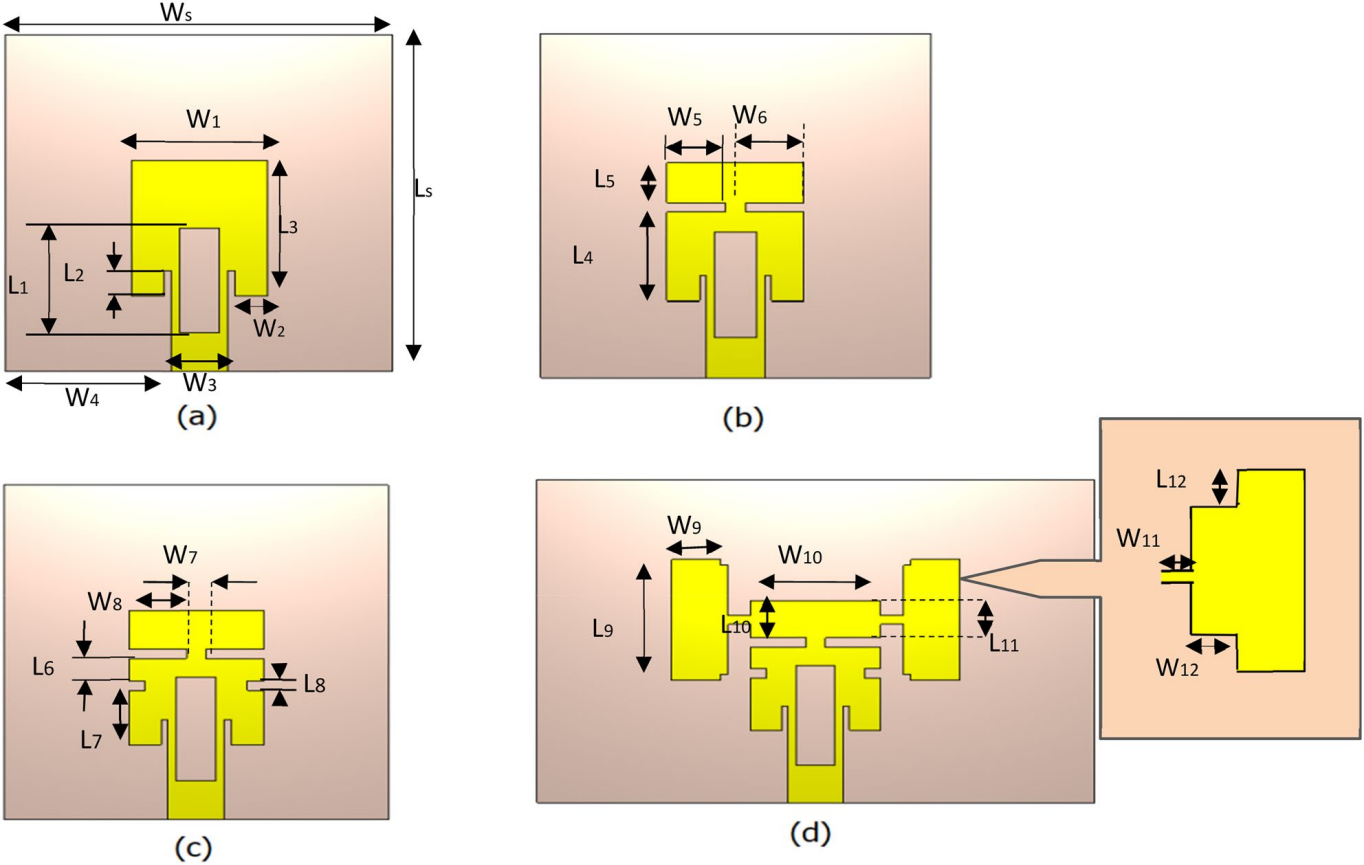
Developmental stages of the proposed antenna; (**a**) Geometry model-a, (**b**) Geometry model-b, (**c**) Geometry model-c, (**d**) Geometry model-d.

**Figure 5:**
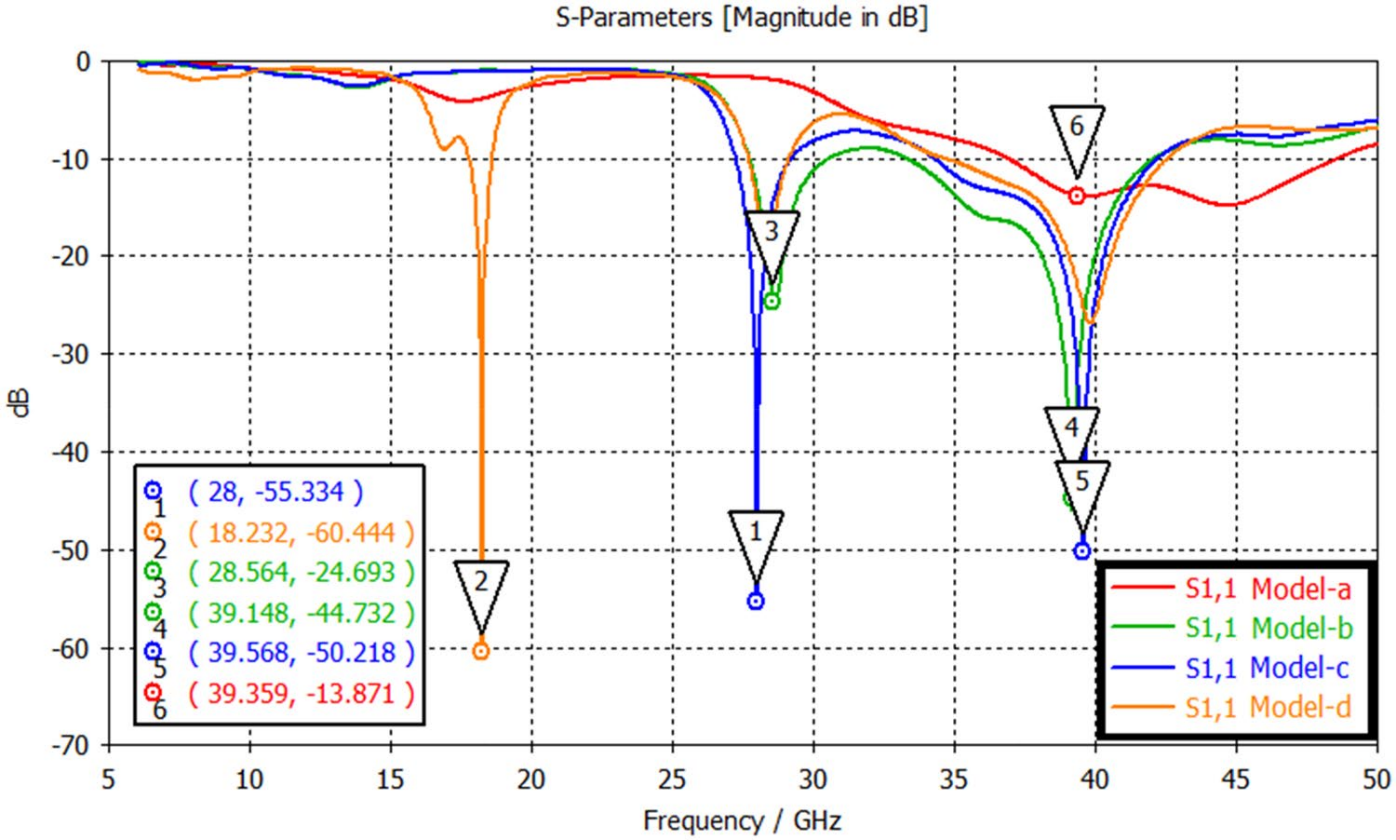
Comparative analysis of proposed designs (model a, b, c & d) showing its S_11_ characteristics.

The antenna structure is designed in four primary stages before reaching the final result. These design stages are named Geometry model-a, Geometry model-b, Geometry model-c, and Geometry model-d shown in Fig. 4. The return loss comparison graph for the design phases is shown in Fig. 5. Table 4 summarizes the design parameters of Reconfigurable Hybrid Antenna.

**Table 4:** Design parameters of reconfigurable hybrid antenna.

Parameter	Parametrical Value (mm)	Parameter	Parametrical Value (mm)	Parameter	Parametrical Value (mm)
L_S._	36	L_8_	1.1	W_3_	3.3
L_1_	11	L_9_	11	W_4_	14.35
L_2_	2.3	L_10_	3.3	W_5_	5
L_3_	14	L_11_	3.3	W_6_	5
L_4_	9.2	L_12_	0.6	W_7_	1
L_5_	3.3	W_S_	32	W_8_	5
L_6_	2.1	W_1_	11	W_9_	6
L_7_	6	W_2_	2.8	W_10_	11
				W_11_	1.2
				W_12_	0.6

When the models in the design stage are examined, the slots opened on the Model-a ensure that the antenna resonates at different frequencies. In addition, thanks to these slots, there has been a reduction in antenna return loss. In addition, thanks to the additional structure added to both sides of the design, the resonating frequency of 18 GHz is achieved.

### Analysis of 18 GHz and 28/38 GHz frequency reconfigurable antenna

The proposed antenna geometry has PIN diodes^40^ and additional patches that act as switching on both sides. Frequency reconfigurability is realized using PIN diodes. When the PIN diodes are “Off”, the antenna resonates at 28 and 38 GHz frequencies. The antenna resonates in the 18 GHz band when the PIN diodes are on. The Simulation of the proposed antenna was carried out using FIT-based simulation elements. In addition, a functional prototype was also produced to verify the simulation results. Return loss was measured using Agilent Technologies PNA-L N5234A Network Analyzer^41^. The antenna is equipped with the advantages of compactness in the specified frequency ranges, ease of manufacture, ability to adjust the frequency, and stable beam width. These makes suggested antenna geometry suitable for 5G short-range wireless systems and K-Band radar applications.

**Figure 6:**
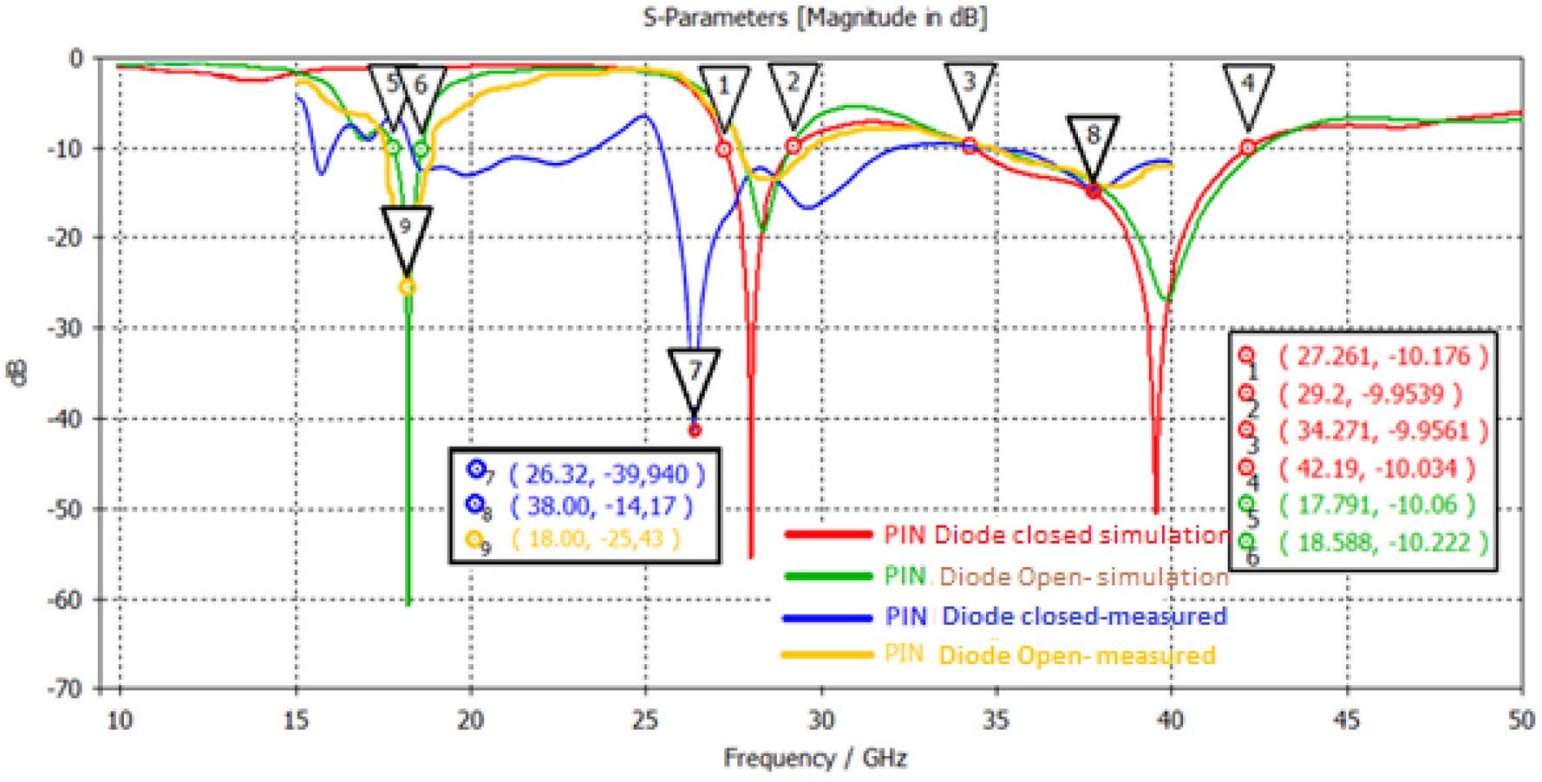
18 GHz and 28/38 GHz frequency reconfigurable antennas’ S_11_ parameter.

In Fig. 6, return loss, the S_11_ parameter under the PIN diode in “On” and “Off” states for the proposed antenna is shown. The bandwidth of the proposed antenna at − 10 dB is 0.79 GHz, 1.94 GHz, and 7.92 GHz, respectively, at frequencies 18.588 GHz, 27.261 GHz, and 34.271 GHz. Also, according to the simulation results, in the 38 GHz, 28 GHz, and 18 GHz frequencies S_11_, the return loss is − 60.68 dB, − 56.38 dB, and − 14.19 dB, respectively. On the other hand, according to the measurement results of the functional prototype fabricated, the frequencies of 18.5 GHz, 28.1 GHz, and 38.1 GHz, S_11,_ the return loss is − 25.44 dB, − 12.5 dB, − 14.2 dB. As can be seen from the results, there are shifts in operating frequencies due to antenna manufacturing tolerances and connector losses. Both PIN diodes were used in the same situation to ensure that the symmetry in the antenna radiation pattern was not disturbed.

### Current and charge distribution

#### Current distribution

It is essential to analyze surface current vector distribution because the distribution of surface current for MFA is vital to achieving the goal. The modified ground plane to alter the current distribution. It has been found that no change in current distribution causes a microstrip patch to produce an vertical radiation pattern. The two-dimensional radiation pattern of single-element antennas does not significantly change due to the current distribution; only antenna arrays are affected. A microstrip patch antenna can produce an end-fire radiation pattern^42^, as shown in Fig. 8, by examining the change in current distribution, which causes minute variations in the radiation pattern.

**Figure 7:**
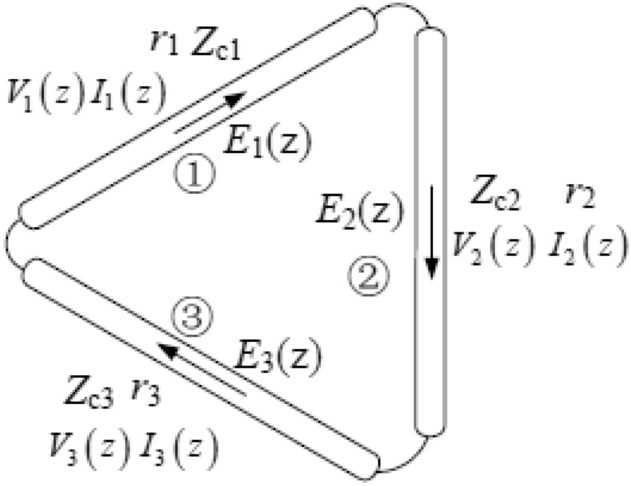
Equivalent circuit transmission line model of the proposed antenna.

Figure 7 shows the matching identical gearbox line model. where the potential and current on the microstripline are denoted by E_i_ (Z) and I_i_ (z), respectively. The propagation constant is ri, while the characteristic impedance is Z_c_i. The tangential element of the incidence electric field is V_i_ (z). The transmission line equations can obtain the current vectors in each section.

The potential and current on the transmission line that are stimulated by the distribution voltage source are determined by the transmission line equations.

14 $$\begin{aligned} \frac{{\partial E_{i} (z)}}{\partial z} &= - j\omega L_{i} \cdot I_{i} (z) + V_{i} (z), \hfill \\ \frac{{\partial I_{i} (z)}}{\partial z} &= j\omega C \cdot E_{i} (z). \hfill \\ \end{aligned}$$

E_i_ (Z) and I_i_ (z) represent the potential and current on the transmission line, respectively. Z_ci_ and r_i_ are the characteristic impedance and propagation constant, respectively. V_i_ (z) is the tangential component of the incidence electric field. The transmission line can obtain the current distribution in each section.

The distributed transmission line parameters are calculated as

15 $$\begin{gathered} L_{i} = \frac{\mu }{2\pi }\left[ {\ln \left( {\frac{{l_{i} }}{\rho }} \right) - 1} \right], \hfill \\ C_{i} = \frac{{\mu_{0} \varepsilon_{0} \varepsilon_{r} }}{{L_{i} }}, \hfill \\ Z_{ci} = \sqrt {\frac{{L_{i} }}{{C_{i} }}} ,\;r_{i} = j\omega \sqrt {L{}_{i}C_{i} } . \hfill \\ \end{gathered}$$

The dielectric resonator (D.R.) theory allows for the calculation of the rectangular D.R. to be performed with a high degree of precision. $TE_{(s + \delta ),m,n}^{x}$ and $TM_{(s + \delta ),m,n}^{x}$ are the primary resonances that are associated with the rectangular D.R. The resonant frequency is determined by applying the formula, $TE_{(s + \delta ),m,n}^{x}$ taking into consideration the mode involved.

16 $$f_{0} = \frac{c}{{2\pi \sqrt {\varepsilon_{r} } }}\sqrt {\left( {\frac{{\left( {s + \delta } \right)\pi }}{a}} \right)^{2} + \left( {\frac{m\pi }{b}} \right)^{2} + \left( {\frac{n\pi }{d}} \right)^{2} } .$$

**Figure 8:**
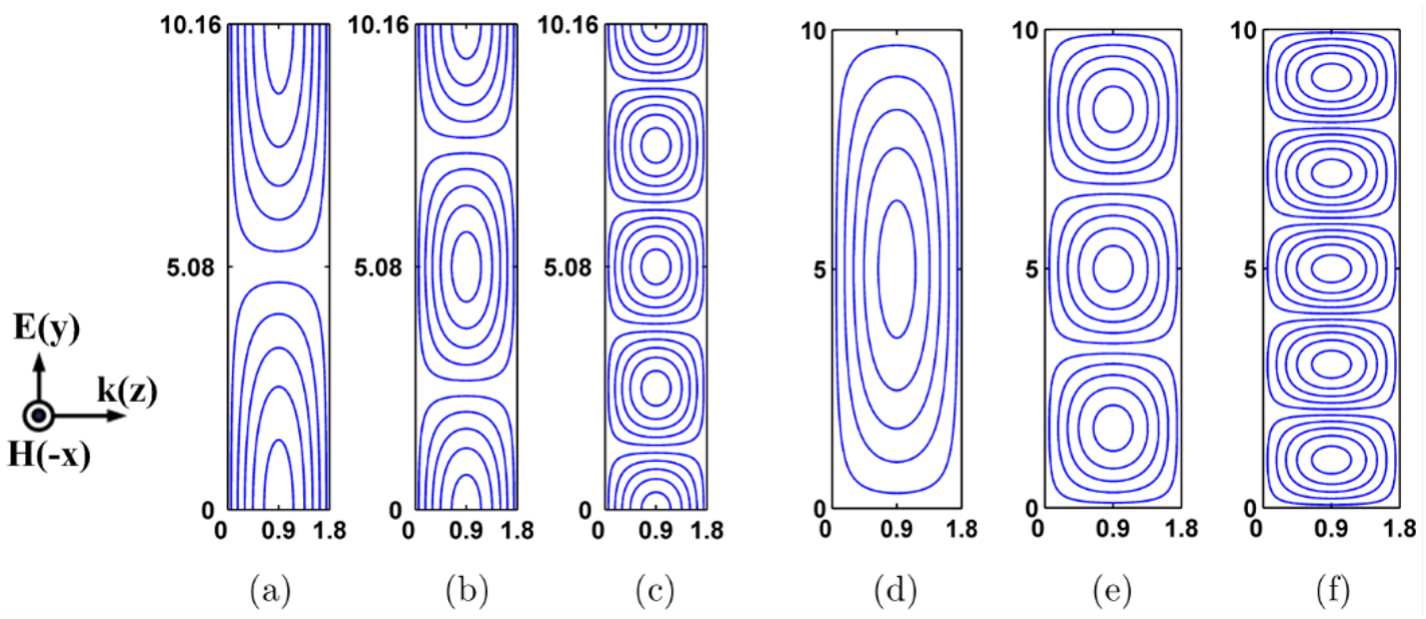
Analysis of modes generated in the proposed 6/18 GHz and 28/38 GHz frequency reconfigurable antenna.

It is possible to compute the resonant frequency of any rectangle D.R. by referring to the equation^37^, which also displays detailed resonant modes. Tuning geometrical size parameters, shapes, and relative dielectric constant can generate resonant modes. Additionally, the boundary condition is a significant aspect that can be utilized to accomplish the modulation of the resonant modes. It is possible to contact the two lateral sides operating in the y-direction on the metallic plates, as demonstrated in Fig. 8a–c. Because of the high permittivity of the D.R.s, these two sides are classified as ideal E-planes, while the remaining four lateral sides are classified as H-planes. It can be shown that the modes shown in Fig. 8a–c are, $TE_{\delta 11}^{x}$, $TE_{\delta 21}^{x}$ and $TE_{\delta 51}^{x}$ respectively. It is impossible to make contact with any of the lateral sides on metallic plates, as demonstrated in Fig. 8d–f. Because of the high permittivity of the D.R.s, these sides ought to be represented by H-planes. In the H-plane, the electric line of force is aligned in a parallel fashion. It can be shown that the modes depicted in Fig. 8d–f are, $TE_{\delta 11}^{x}$, $TE_{\delta 31}^{x}$ and $TE_{\delta 41}^{x}$ respectively. The rectangular D.R. exhibits a variety of resonant modes, which in turn results in various electromagnetic responses, regardless of whether or not there is contact on the metallic plate. As a result, boundary conditions have the potential to be an effective instrument for modulating the transmission characteristic of the reconfigurable hybrid antenna.

**Figure 9:**
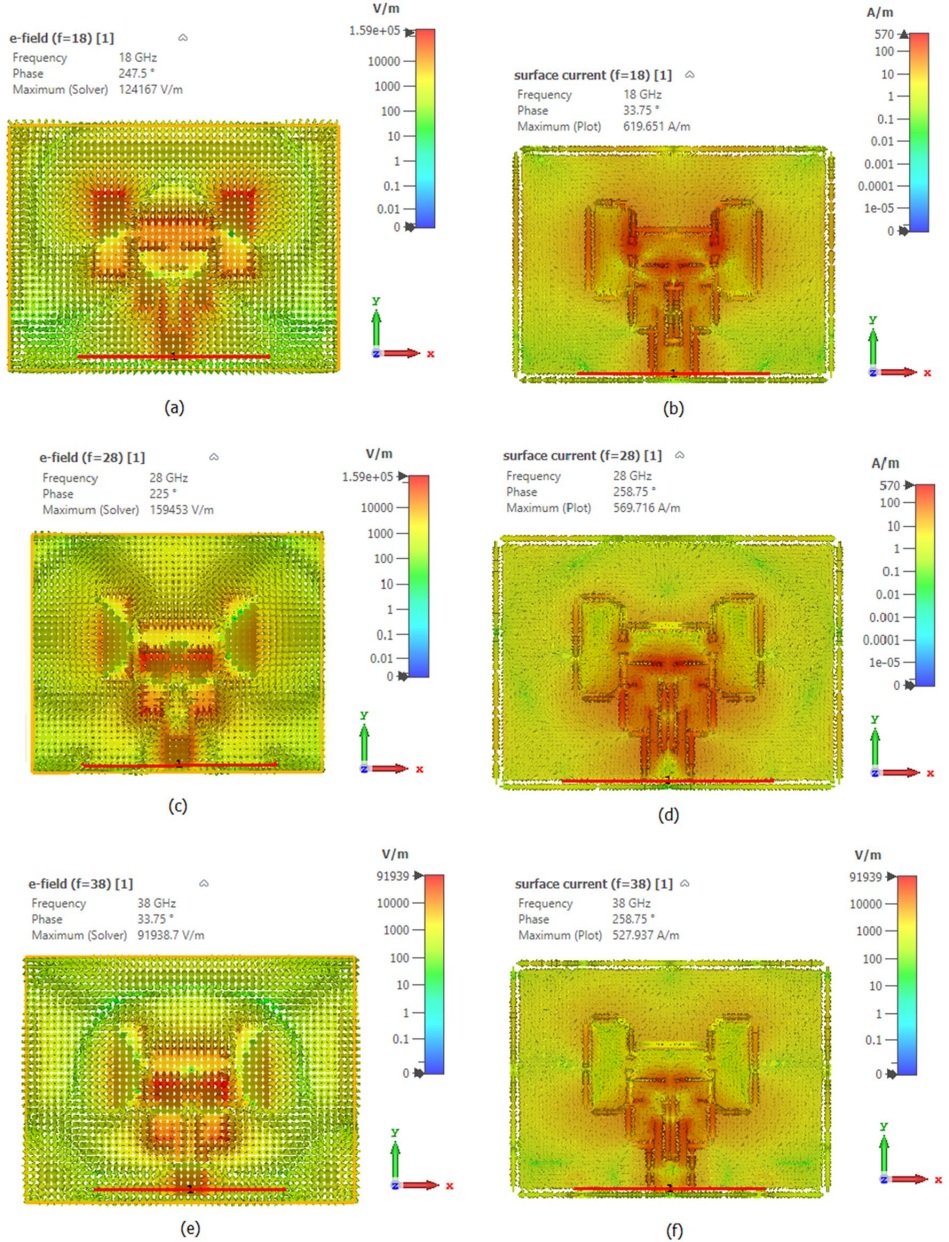
Frequency reconfigurable antenna structure (**a**) 18 GHz Electric field distribution, (**b**) Distribution of surface current at 18GHz, (**c**) Distribution of Electric field at 28GHz, (**d**) 28 GHz Surface current distribution, (**e**) 38 GHz Electric field distribution, (**f**) 38 GHz Surface current distribution.

Figure 9a shows the electric field distribution at 18 GHz resonant frequency, while Fig. 8b shows the surface current distribution at 18 GHz resonant frequency. The electric field distributions at 18 GHz are set in the 0–124,167 V/m range. The surface current vector distribution at 18 GHz was achieved in the 0–619.65 A/m range. Figure 9c shows the electric field distribution at the frequency of 28 GHz, and Fig. 9d shows the distribution of surface currents. The electric field distributions at 28 GHz were adjusted in the range of 0–159,453 V/m, while the surface current distribution was achieved in the 0–569.71 A/m range. Figure 9e shows the distribution of surface currents at the resonant frequency of 38 GHz, as shown in Fig. 9f. The electric field distributions for 38 GHz were set in the range of 0–91,938.7 V/m, while the surface current distribution was achieved in the 0–527.93 A/m range. The ground plane, upper and lower patch surfaces, and the patch will all create a charge distribution when electrifying the patch. Figure 9 displays the charge distribution for the EHA and conventional methods on the antenna surface.

### Gain performance analysis of 6/18 GHz and 28/38 GHz frequency reconfigurable antenna

Moreover, the gain of the selected approach over the old model is performed at varying frequency levels. In addition, the developed method has a significant gain (1.7257) compared to the conventional method like EHO. As a result, the suggested model’s superiority over the other standard approach is established.

**Figure 10:**
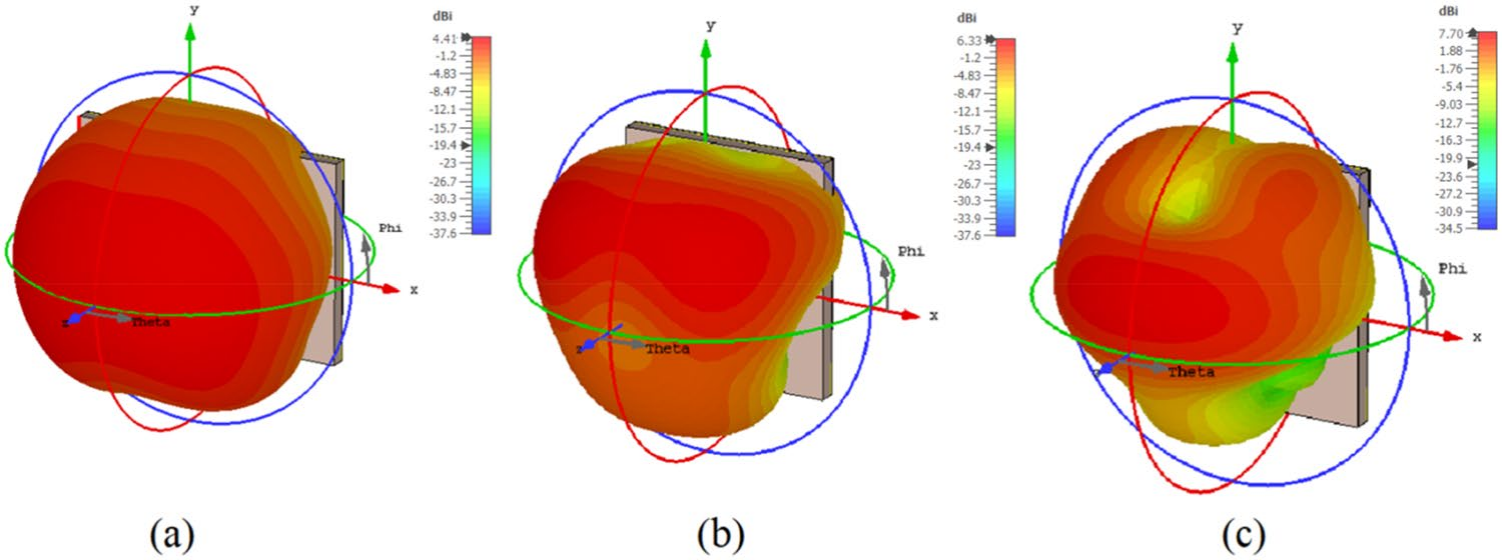
6/18 GHz and 28/38 GHz frequency reconfigurable antenna 3D radiation pattern (**a**) 18 GHz; (**b**) 28 GHz; (**c**) 38 GHz.

**Figure 11:**
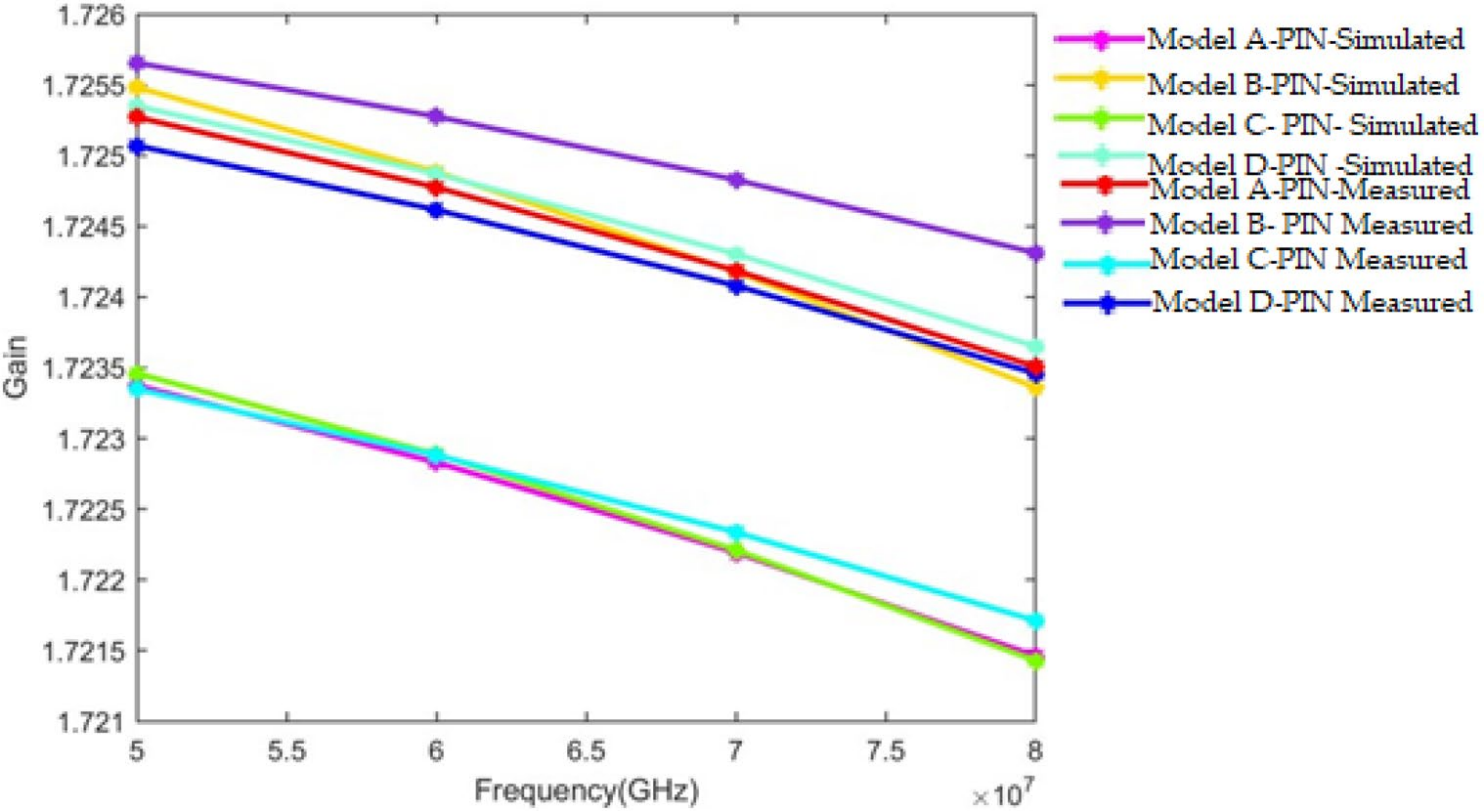
Gain response analysis of proposed antenna under PIN diode operating condition.

Figure 10 shows the 3D radiation pattern of the proposed design. In addition, according to the proposed frequency reconfigurable antenna simulation results, it provides a gain of 4.41 dBi, 6.33 dBi, and 7.70 dBi at 18 GHz, 28 GHz, and 38 GHz, respectively, as shown in Fig. 11.

**Figure 12:**
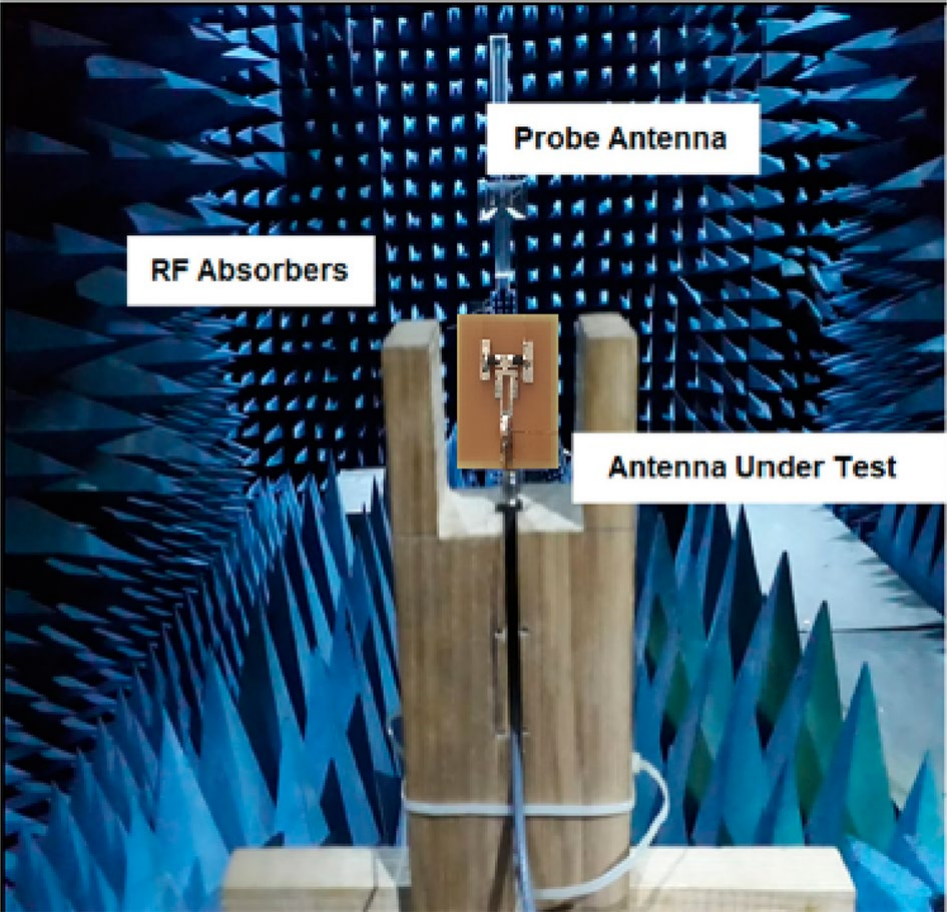
Measurement setup of proposed antenna in Anechoic chamber.

The suggested antenna structure was designed, simulated, and analyzed using HFSS. The PIN diode allows frequency customization and several antenna modes. Each antenna mode operates in a distinct frequency band. Figure 12 displays the prototype’s photos and measurement setup for pattern reconfiguration inside each mode and frequency region. Table 5 shows the performance analysis of the proposed antenna with other related works.

**Table 5:** Performance analysis of the proposed antenna with other relevant designs.

Ref. no.	Size (Ls × Ws)	No. of switches	Types of switches	No. of operating frequency bands	Max. number of beams	Bandwidth (MHz)	Peak gains (dBi)
^24^	40 × 33	6	Pin diode	3	2	180/254/455	2.24/2.85/3.3
^25^	41 × 37	4	Varactor	2	3	220/450	4.01/3.35
^26^	112 × 108	8	Pin diode	2	3	1745/1525	3.8/3.6
^27^	45 × 38	6	Varactor	3	3	265/452/350	2.24/2.76/3.4
^28^	148 × 154	4	Pin diode	2	3	725/455	6.2/6.6
The Proposed Work	81 × 55	2	Pin diode	6	3	797/1939/7919	4.41/6.33/7.70

## Design of ECSRR bow-tie antenna

**Figure 13:**
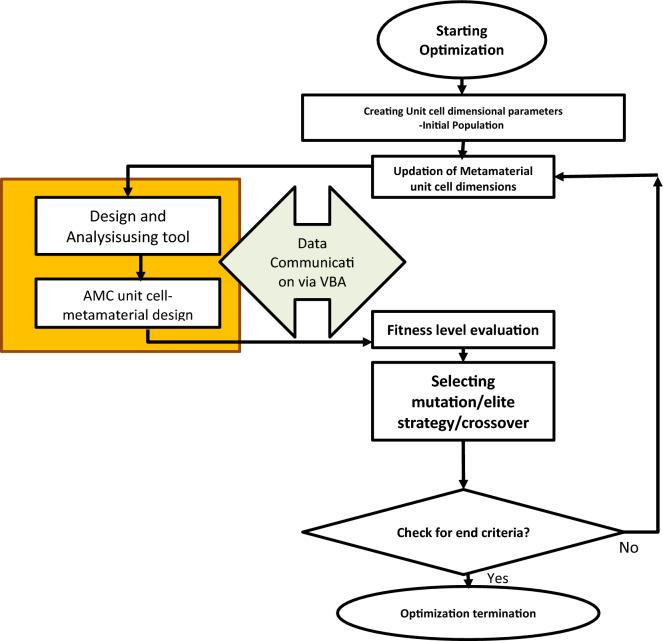
Genetic algorithm flowchart for the optimization of antenna geometrical parameters.

The proposed ECSRR bow-tie microstrip patch antenna consists of one ECSRR bow-tie element, one microstrip line bow-tie element and microstrip line feeding. The practical design dimensions of the proposed ECSRR bow-tie antenna are achieved by optimization through a genetic algorithm, as shown in Fig. 13. The dimensions of the ECSRR bow-tie antenna antenna are shown in Table 6.

**Table 6:** Parametrical dimensions of ECSRR BOW-TIE Antenna.

Parameter	Dimension (in mm)	Parameter	Dimension (in mm)
W_1_	28	L_3_	7
W_2_	3.1	L_4_	7.9
W_3_	0.9	L_5_	4
W_4_	5.1	L_6_	1
L_1_	21	L_7_	1.3
L_2_	7.9		

**Figure 14:**
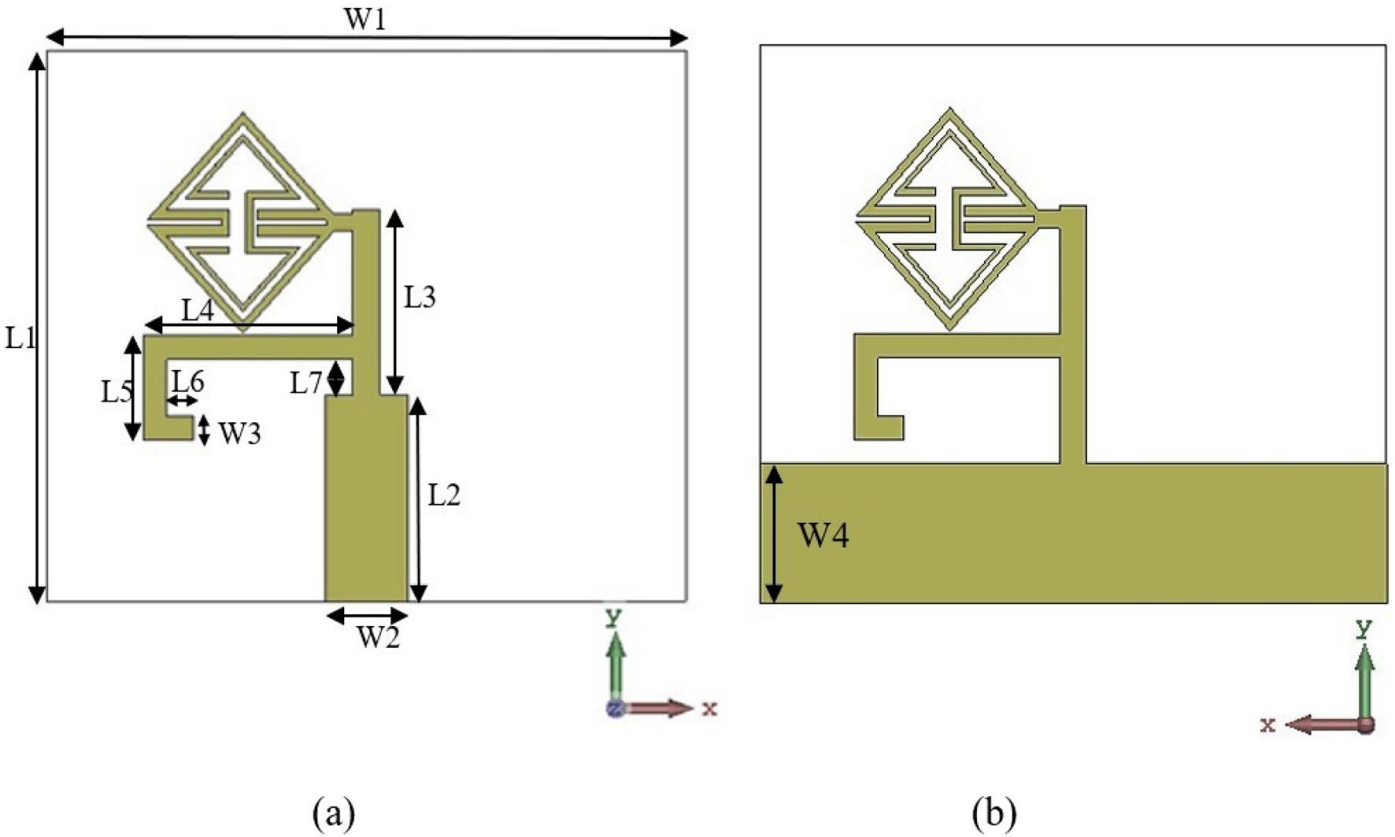
Structure ECSRR bow-tie antenna.

The suggested ECSRR bow-tie antenna structure is shown in Fig. 14. The bow-tie component has two “arms”, one printed on the dielectric substrate’s top and bottom layers. In a triangle shape, the ECSRR metamaterial unit cell structure^25^ and the microstrip stub are used in the bow-tie antenna. The suggested bow-tie antenna design receives its electricity via microstrip line feeding.

### Simulation of ECSRR bow-tie antenna

**Figure 15:**
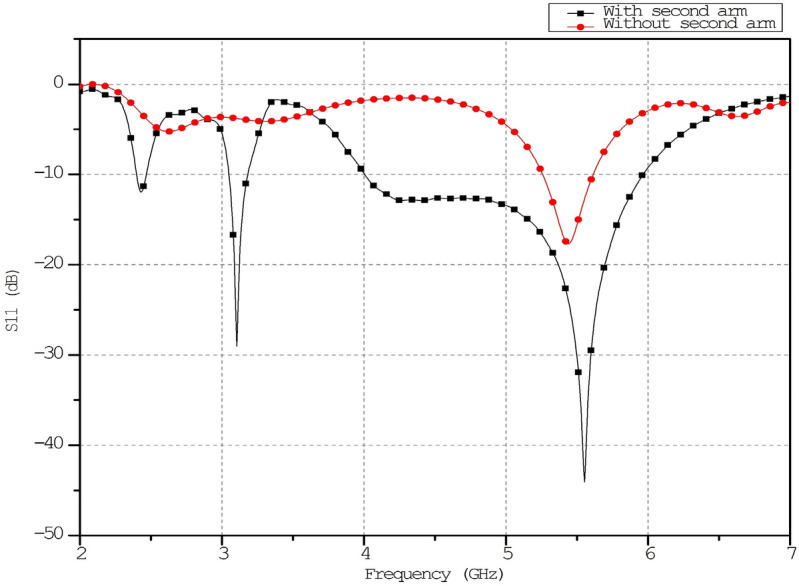
Simulated return loss of the ECSRR bow-tie antenna.

The substrate’s bottom layer uses the comprehensive ground plan and microstrip-fed ECSRR bow-tie antenna. The bow tie uses ECSRR unit cell construction. The first component matches impedance at 5.5 GHz. Optimizing the second arm length and ECSRR structure size increases bandwidth from 4 to 6 GHz. The ECSRR bow-tie antenna measures 28 by 21 mm. Figure 15 shows the ECSRR bow-tie antenna’s simulated return loss.

**Figure 16:**
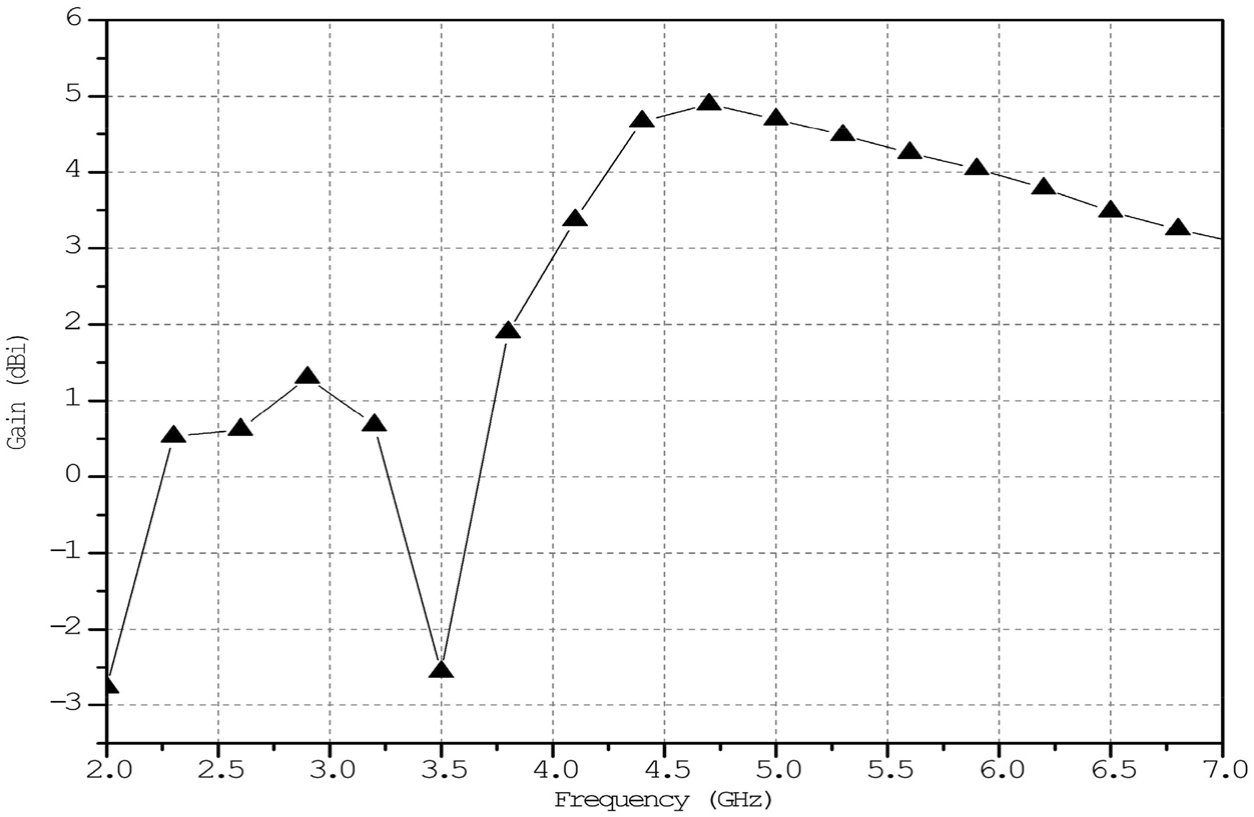
ECSRR Bow-Tie antenna’s simulated gain.

Figure 16 shows the Simulation of ECSRR bow-tie antenna gain. At 4.7 GHz, the modelled ECSRR bow-tie antenna gains 4.8 dBi. The antenna gains 2.9, 4.7, 4.35, and 4 dBi at 4–6 GHz. The antenna gains 2.9, 4.7, 4.35, and 4 dBi at 4–6 GHz. The antenna is 28.2 × 20.5 × 1.6 mm. The antenna’s revolutionary design creates its focused emission pattern and smaller size.

When the ECSRR metamaterial is placed over the dielectric substrate at a low height, it causes the conventional patch antenna to experience parasitic loading. Parasitic loading results in proximity coupling between the substrate and patch antenna, forming an electromagnetically coupled system. The interaction of electromagnetic fields between the patches and metamaterial substrate leads to an enhancement in the gain of the composite system. The proposed antenna can achieve augmentation of return loss (S_11_) by utilizing the cavity effect due to the second arm of the ECSRR bow-tie antenna structure.

### Enhancing gain of ECSRR bow-tie antenna withECSRR metamaterial unit cell

The triangular ECSRR metamaterial^26^ boosts the bow-tie antenna’s gain with the help of triangle-shaped ECSRR metamaterial unit cells. The wire-shaped strip and triangle-shaped split rings are printed on the dielectric substrate. An end-fire array-radiating ECSRR bow-tie antenna is proposed.

Triangle-shaped ECSRR periodical metamaterial unit cells increase ECSRR bow-tie antenna end-fire gain. ECSRR Bow-tie antennas have a triangle-shaped 3 × 4 metamaterial unit cell array. Three triangle-shaped unit cells are needed for end-fire antenna coverage.

**Figure 17:**
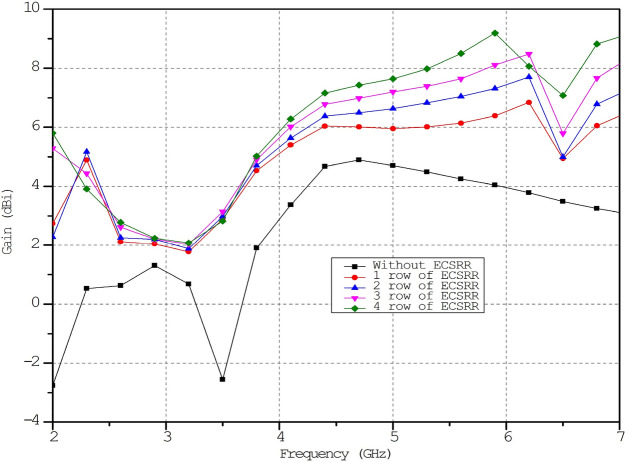
Comparative gain analysis of ECSRR bow-tie microstrip patch antenna with ECSRR-based metamaterial unit cells and without ECSRR-based metamaterial unit cells.

The column has three cells per unit. The effect of changing the ECSRR metamaterial’s unit cell array’s row count has been parametrically examined. Figure 17 compares ECSRR unit cell gain improvement with and without it. The antenna measures 28 × 49 mm. Simulations show a maximum gain of 9.2 dBi for the proposed antenna. ECSRR metamaterial unit cells boost antenna gain by 5.14 dBi at 5.9 GHz. A 3 × 4 metamaterial unit cell array enhances this. Analysis reveals that antenna gain increases with frequency range.

**Figure 18:**
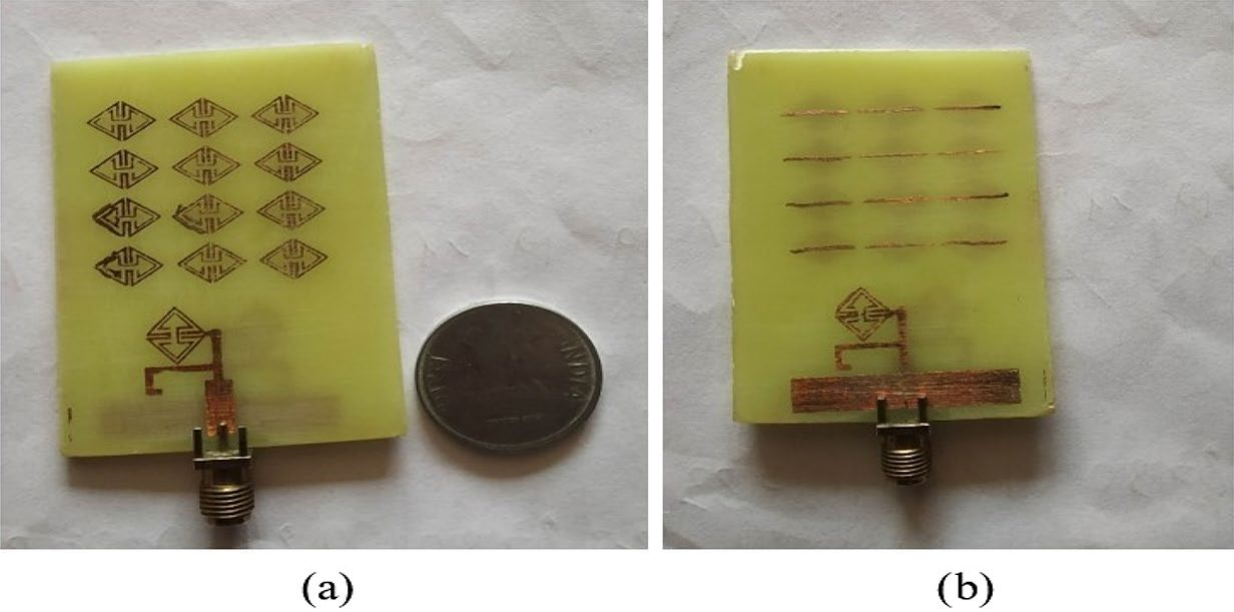
Prototype model of ECSRR metamaterial unit cells (Bow-tie antenna) (**a**) top layer, (**b**) bottom layer.

**Figure 19:**
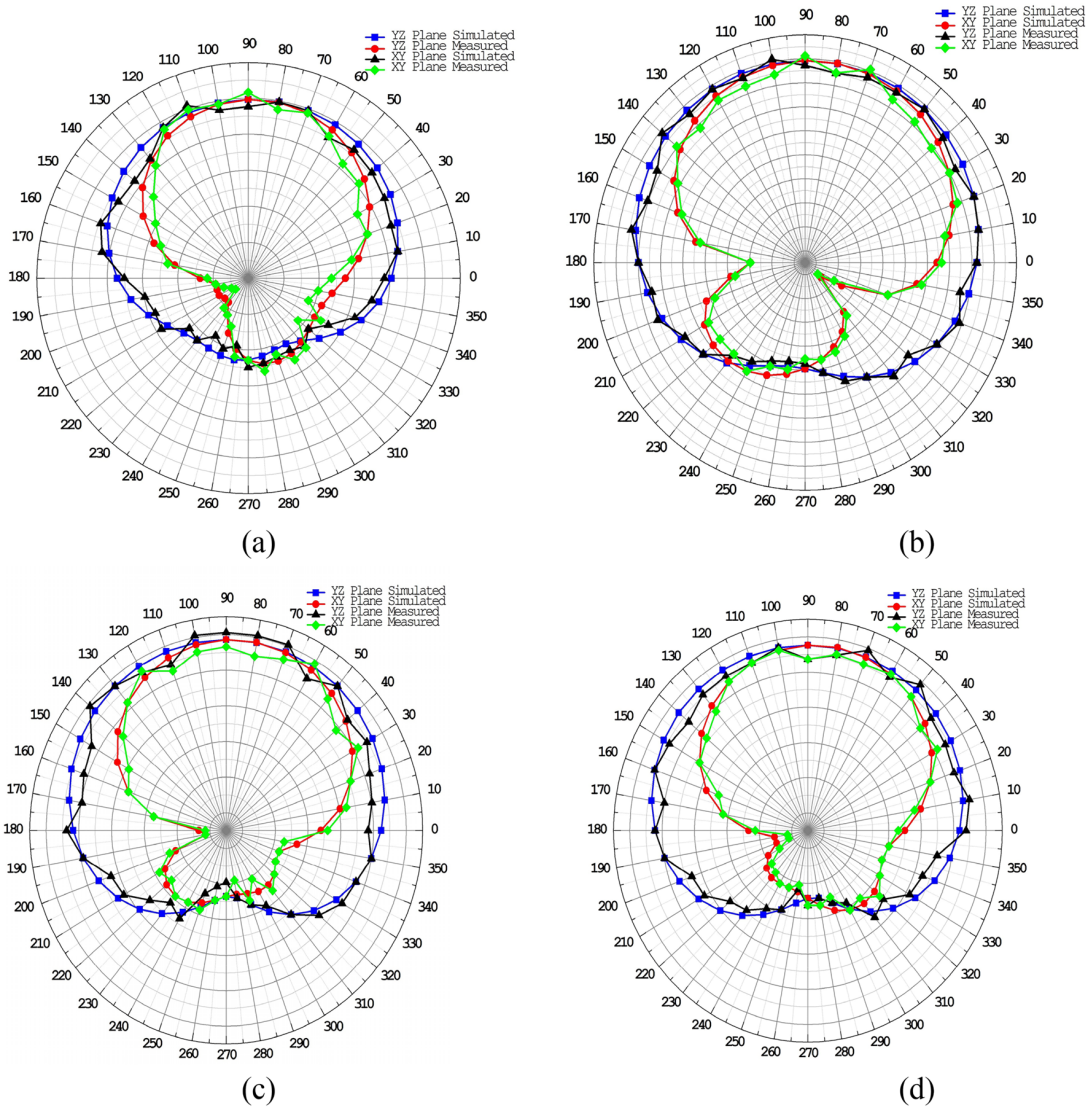
Comparative analysis of measured and simulated ECSRR bow-tie antenna radiation pattern with metamaterial unit cells resonating at (**a**) 3.49 GHz. (**b**) 4.4 GHz. (**c**) 5.46 GHz. (**d**) 6.17 GHz.

The proposed ECSRR metamaterial-based bow-tie antenna yields a gain of 6 dBi at 4 GHz and 7.3 dBi at 5 GHz. 8.39 dBi at 5.44 GHz and 8.89 dBi at 6.15 GHz. According to the research, proposed metamaterial structure boosts bow-tie antenna gain at 4 GHz to 6 GHz. ECSRR bow-tie antenna prototype in Fig. 18. Figure 19 displays the observed and simulated 2D radiation pattern of the ECSRR bow-tie microstrip patch antenna in the Y–Z and X–Y planes at various frequencies within its operational band.

The advent of so-called metamaterials (MTMs), manmade materials with exotic characteristics and manufactured electromagnetic responses not often found in nature, has allowed for an alternate design approach^11–13^. Numerous radiating and scattering systems now have better performance characteristics as a result of this. Preliminary analytical research on the metamaterial-based ECSRR systems presented in Refs.^14,15^ suggests ECSRR system with a dimensionally compact half-wave dipole antenna that radiates in the presence of either ENG or DNG.

Compared to an isotropic antenna, the suggested ECSSR antenna has a negative dBi gain, indicating that it does not radiate particularly well in that particular direction. Always remember that gain and directivity are three-dimensional functions dependent on azimuth and elevation angles. A directional antenna can have a high gain in one direction but a low gain in the other direction. The pattern form, a bad match, internal losses, and external stress may contribute to the poor gain.

## Reduction of mutual coupling using ECSRR EBG structure

### Two-element ECSRR bow-tie antenna simulation

**Figure 20:**
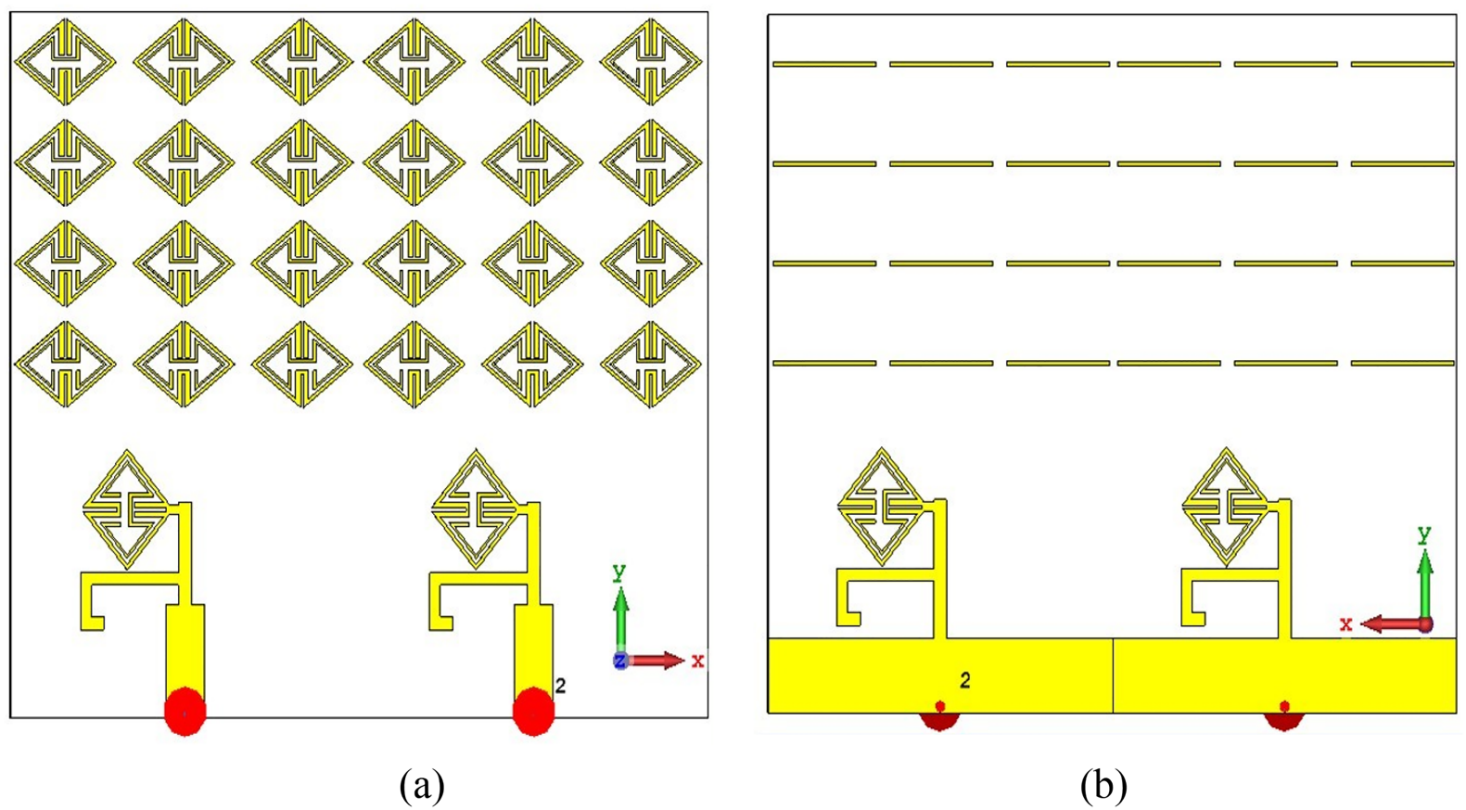
Two-element ECSRR bow-tie antenna (**a**) top view, (**b**) bottom view.

**Figure 21:**
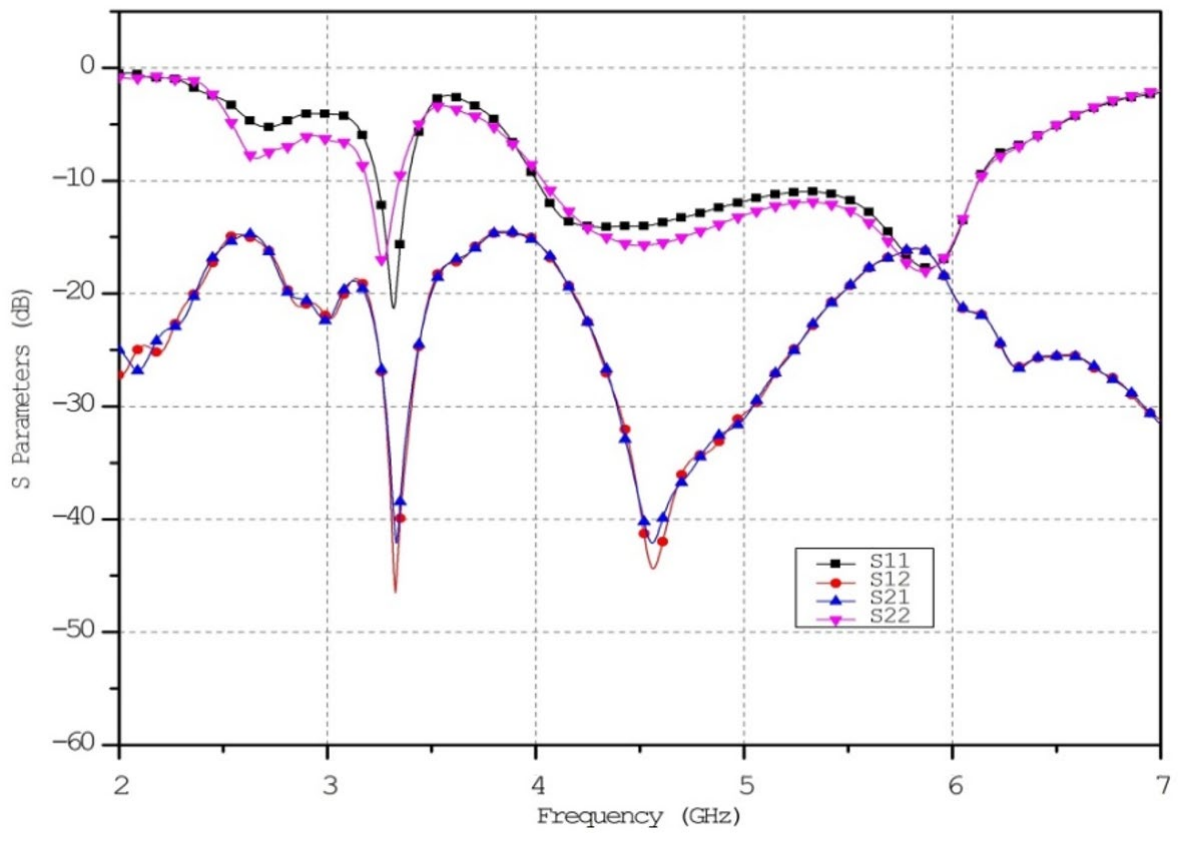
Simulated reflection coefficient (S_11_) parameters of two-element ECSRR bow-tie antenna.

The metamaterial ECSRR bow-tie shaped microstrip patch antenna is shown in Fig. 20 using a metamaterial structure and a two-element design, which measures dimensions of 56 mm by 49 mm. Figure 21 displays the calculated scattering parameters. From 4.45 to 6.15 GHz, the simulated bandwidth of 2.15 GHz. Within the 4.15 GHz to 5.5 GHz range, the mutual coupling of the two antennas is less than − 20 dB, and at all other frequencies, − 15.1 dB and − 20.2 dB. The Multiple Antenna System relies heavily on the ECC, derived from the relationship between the radiation patterns of the various antennas.

**Figure 22:**
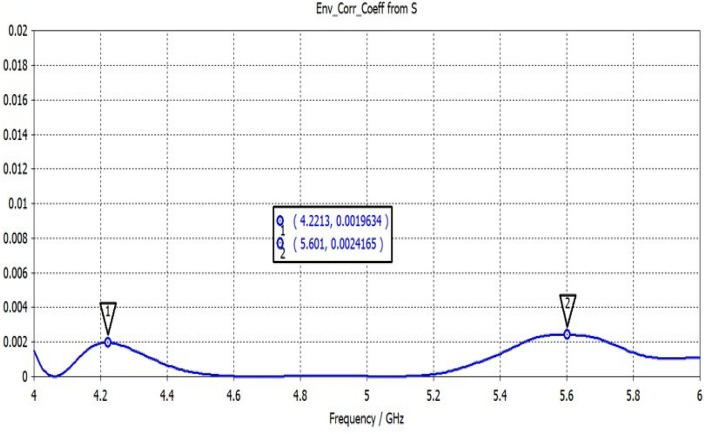
ECC of a two-element ECSRR bow-tie antenna.

Figure 22 shows the ECC determined for the two-elemental ECSRR-based bow-tie antenna. The ECC is less than 0.0025 over the 4–6 GHz operating frequency range. The mutual coupling is reduced by placing the ECSRR-based Electromagnetic Band Gap structure separating the antenna elements.

### Simulation of two elements of ECSRR bow-tie antenna with ECSRR EBG structure

**Figure 23:**
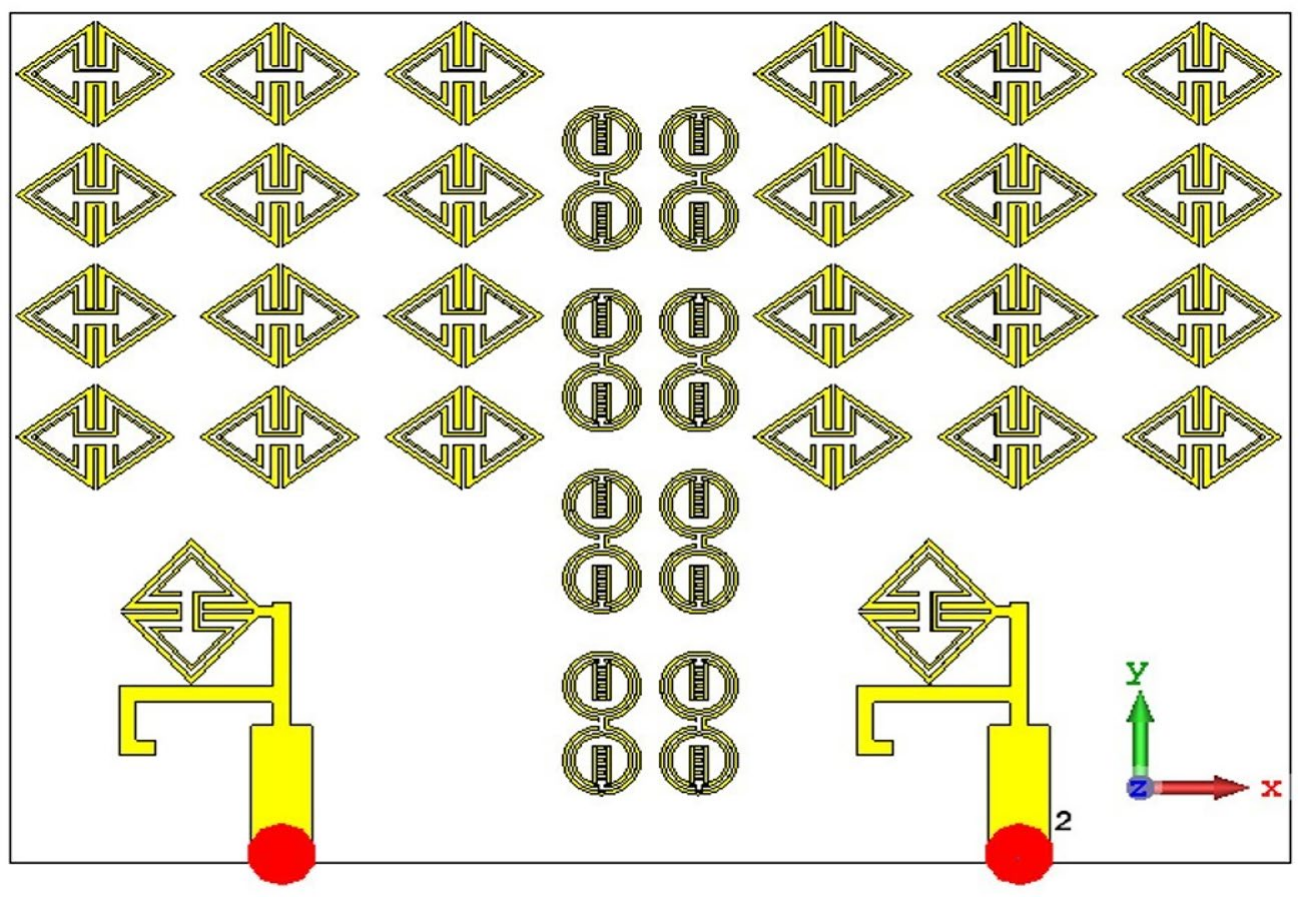
ECC of a two-element ECSRR bow-tie antenna top layer.

**Figure 24:**
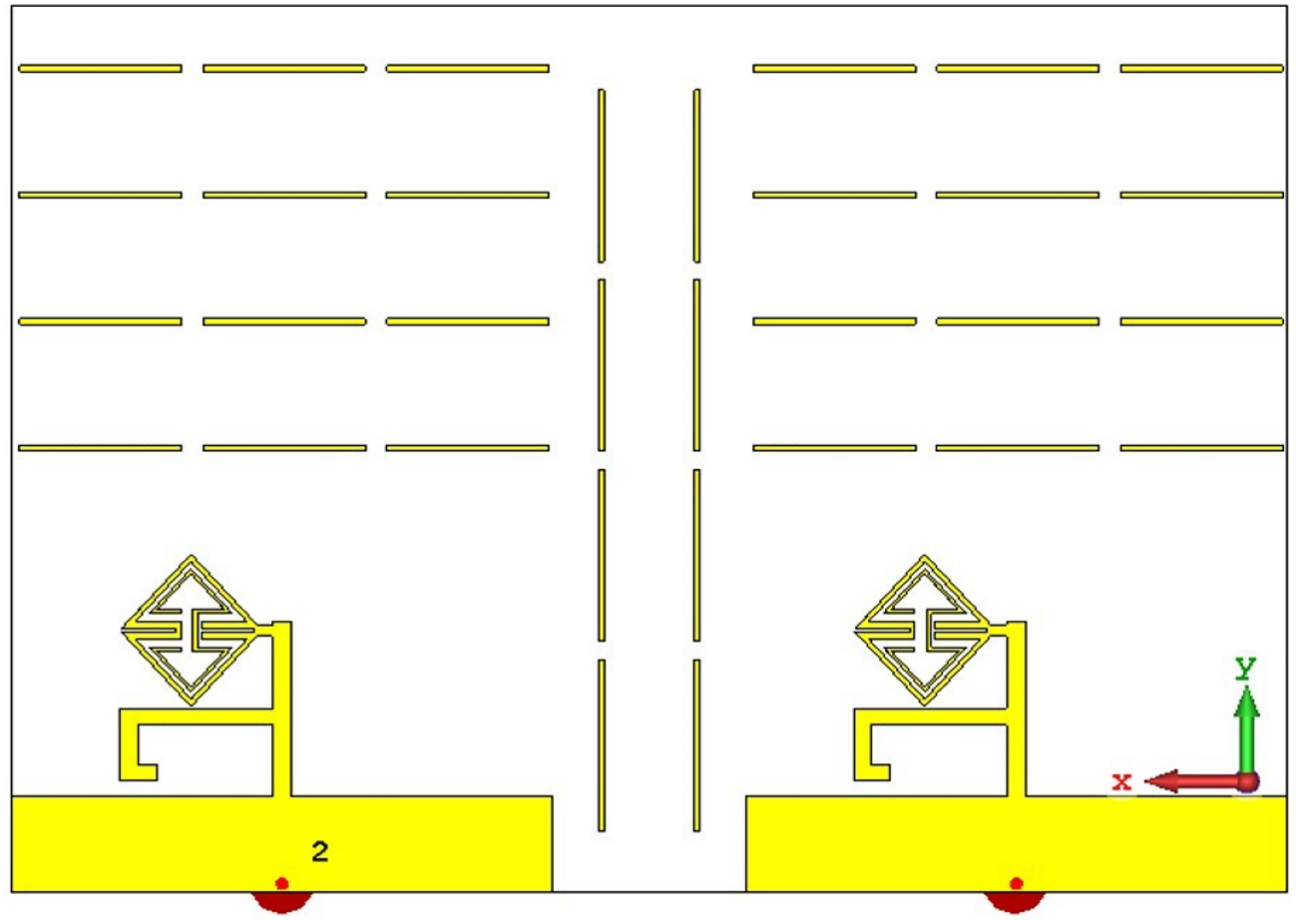
ECC of a two-element ECSRR bow-tie antenna bottom layer.

Figures 23 and 24 depict the ECSRR EBG periodic structure interposed between the two elements of the ECSRR-based bow-tie antenna and the ECSRR metamaterial structure. The bow-tie microstrip patch antenna has a length of 66 mm and a width of 49 mm.

**Figure 25:**
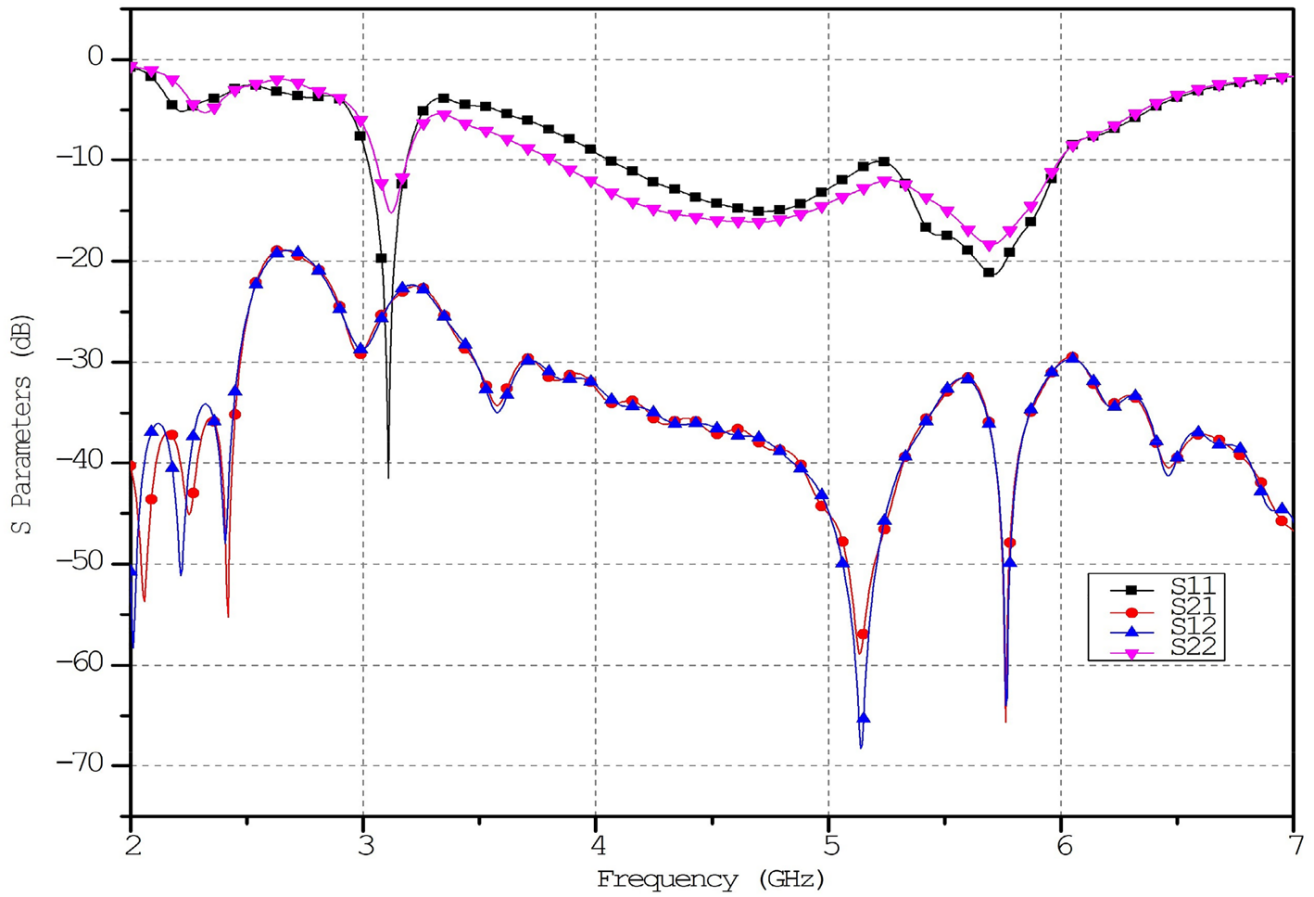
Simulated reflection co-efficient (S_11_) of ECSRR bow-tie antenna with ECSRR EBG periodic structure.

A periodic ECSRR EBG structure is sandwiched there at a 2 × 4 period to minimize interference between the antenna’s elements. Scattering parameters for the ECSRR EBG periodic structure as modeled by the ECSRR bow-tie antenna with two elements are shown in Fig. 25. Results from computer simulations show that the mutual coupling existing between the bow-tie antenna’s two elements is reduced thanks to the ECSRR EBG’s periodic structure. Overall, the mutual coupling is less than − 30 dB across the whole frequency range of operation. At 5.15 GHz, isolation is at its highest point, at − 68 dB.

The addition of the metamaterial ECSSR resonator does not impact the formation of compact co-polarization patterns in either of the two scenarios. On the other hand, compared to the traditional bow-tie polarised antenna, we can achieve a significant improvement in cross-polarization discrimination. The Simulation demonstrates a 12 dB improvement in cross-polarisation discrimination compared to the traditional antenna. Figure 24 illustrates the mutual coupling and reflection coefficients between two orthogonal ports regardless of whether or not S-RR inclusions are present. It has been observed that the mutual coupling decreases by 2 dB, and the return loss for the input port are S_11_ = − 42 dB at the center frequency. This is the case.

**Figure 26:**
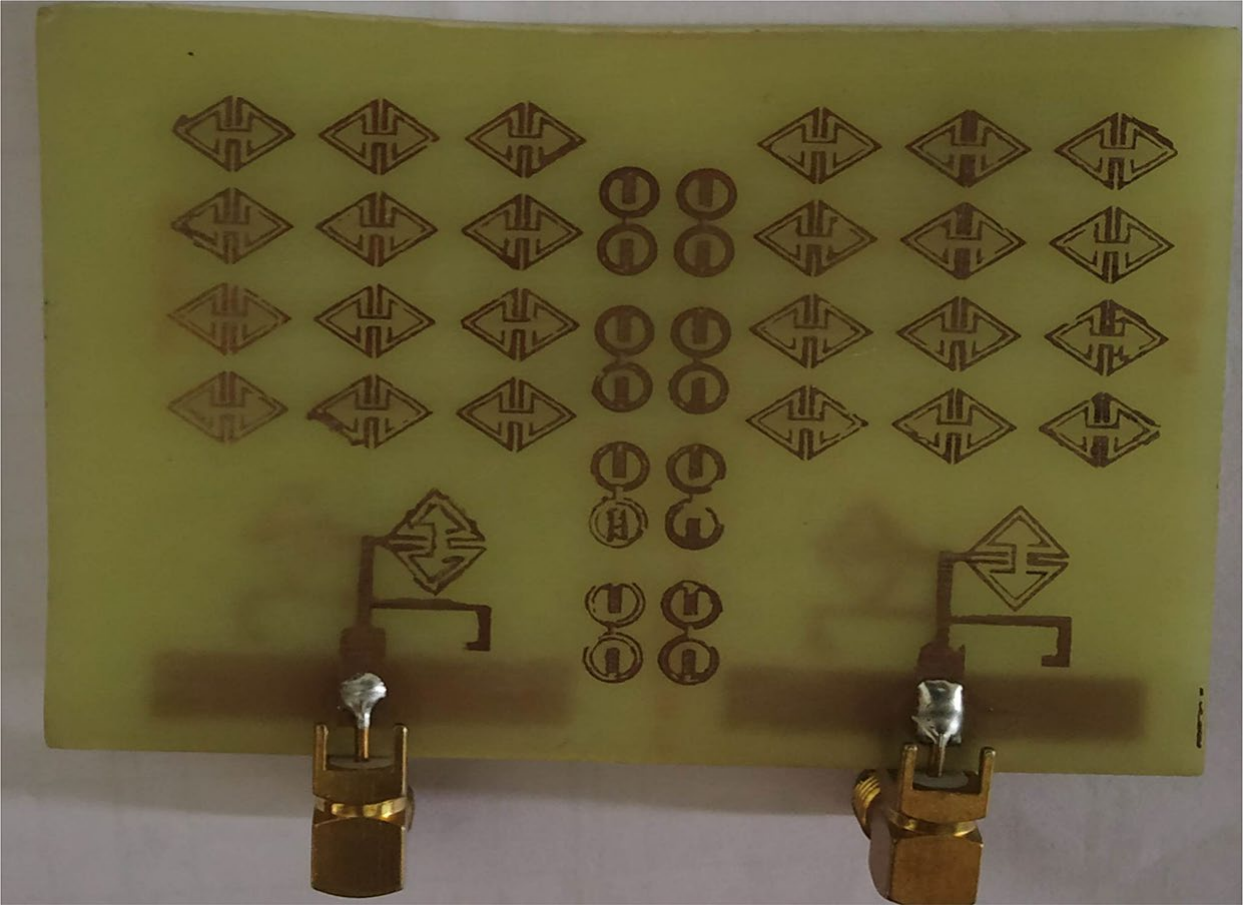
Prototype model of two-element ECSRR bow-tie antenna with ECSRR EGB structure top layer.

**Figure 27:**
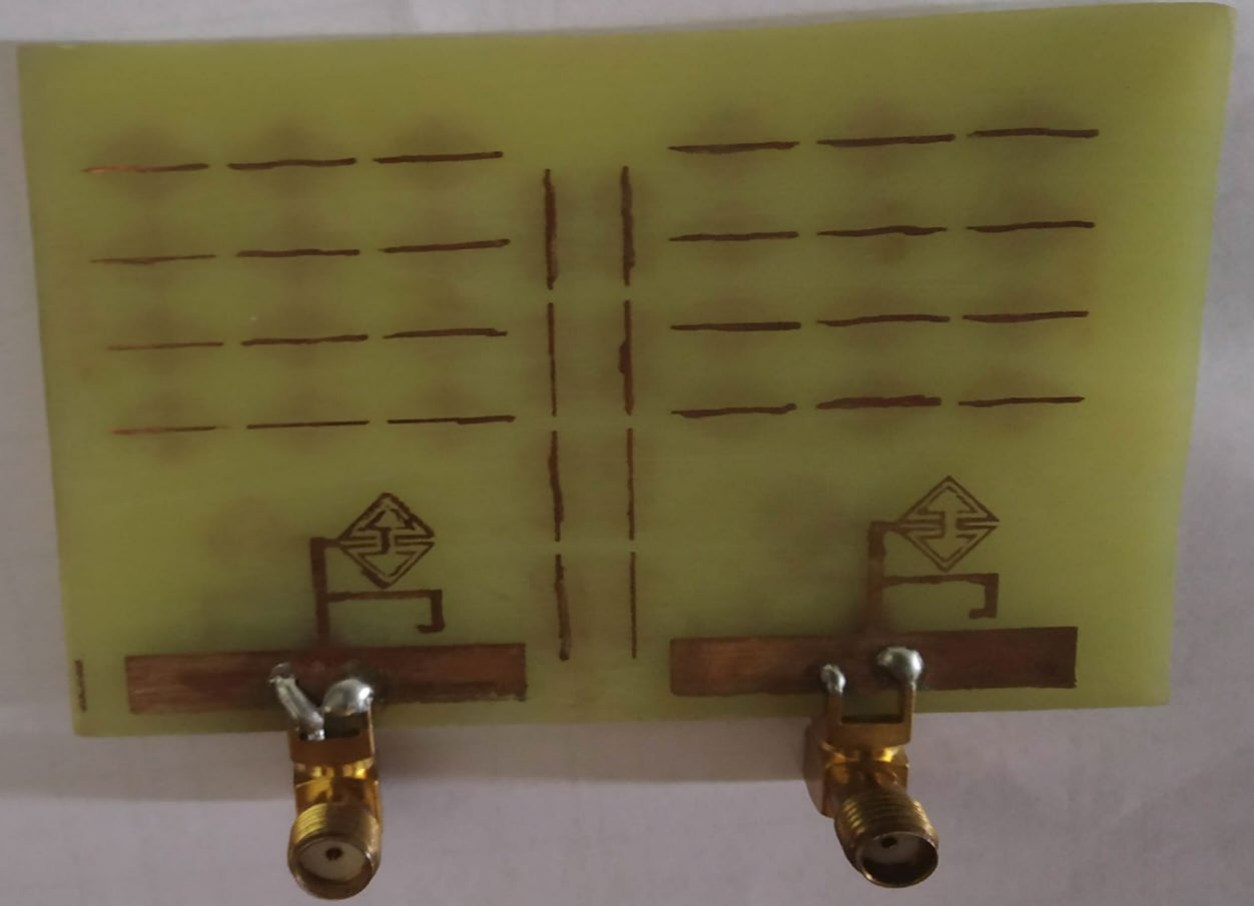
Prototype model of two-element ECSRR bow-tie antenna with ECSRR EGB structure bottom layer.

**Figure 28:**
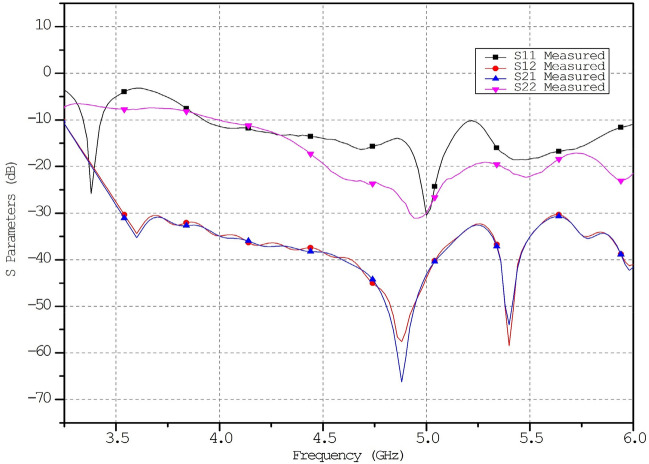
Measured reflection coefficient (S_11_) of ECSRR bow-tie antenna with ECSRR EBG structure.

Figures 26 and 27 depict the two-element ECSRR bow-tie antenna prototype model built using the ECSRR EBG structure, and Fig. 28 displays the scattering parameters that were measured. The measured result demonstrates that mutual coupling is less than − 32 dB across the frequency operating band. At 4.9 GHz, the isolation is a peak of 66 dB.

The slight variation in simulated and experimental results is because of the following reasons.

Simulations often rely on mathematical models that make certain assumptions to simplify the analysis. These assumptions may not perfectly reflect real-world conditions. Simplifications in geometry, material properties, or environmental factors can contribute to differences between simulated and experimental results. The actual fabrication process of an antenna may introduce variations in dimensions and material properties. The connection between the antenna and the measurement equipment can introduce impedance mismatches or signal losses not considered in the Simulation. The characteristics of the feed lines and connectors may differ from the simulated model. Calibrations and measurement uncertainties can impact the accuracy of the experimental results.

**Figure 29:**
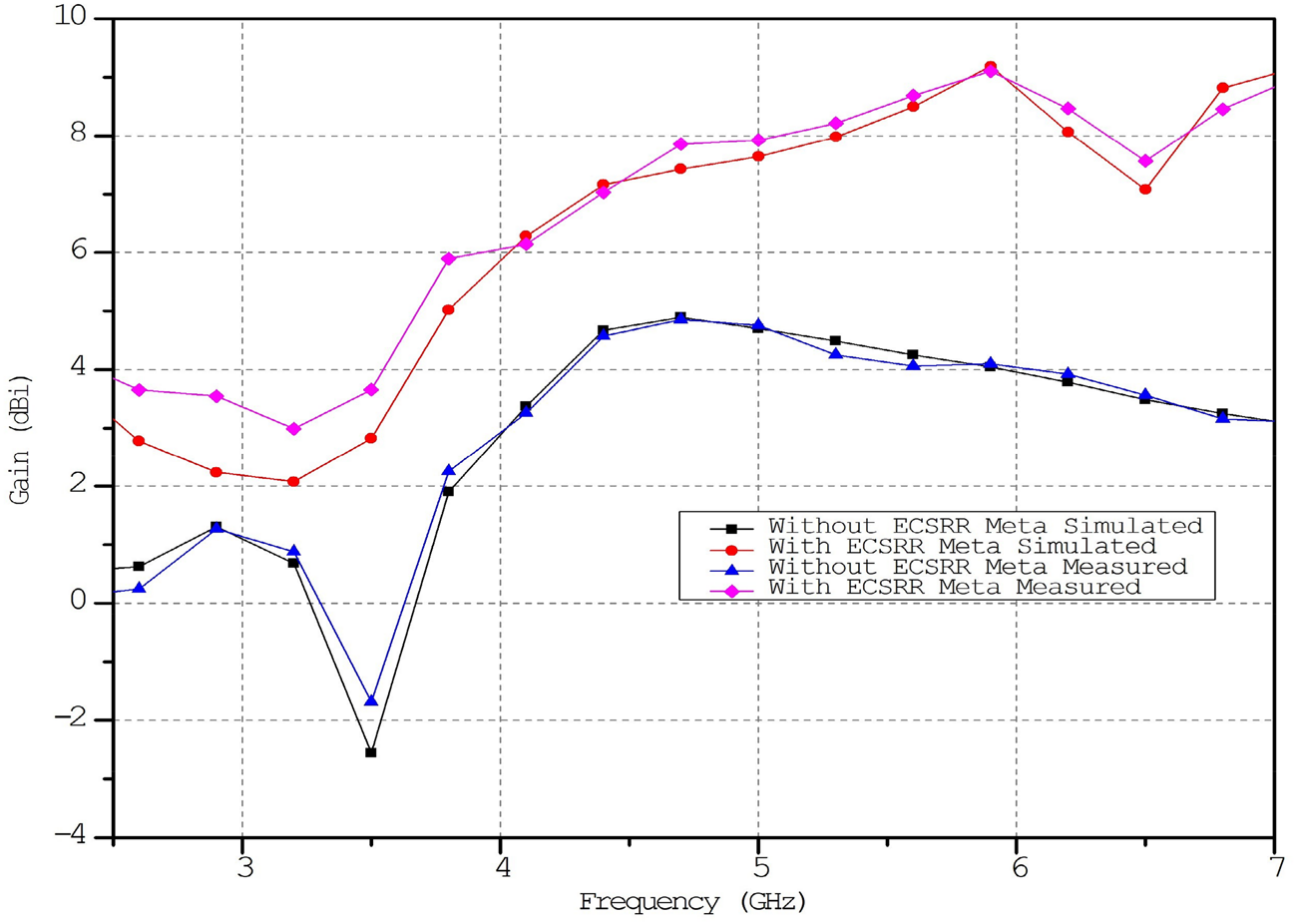
Measured and Simulated gain of ECSRR bow-tie antenna without and with ECSRR metamaterial unit cells.

**Figure 30:**
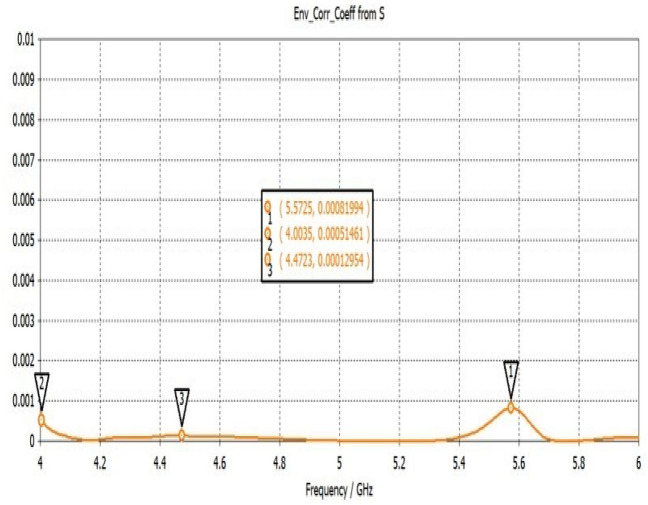
ECC of ECSRR bow-tie antenna with EBG structure.

**Figure 31:**
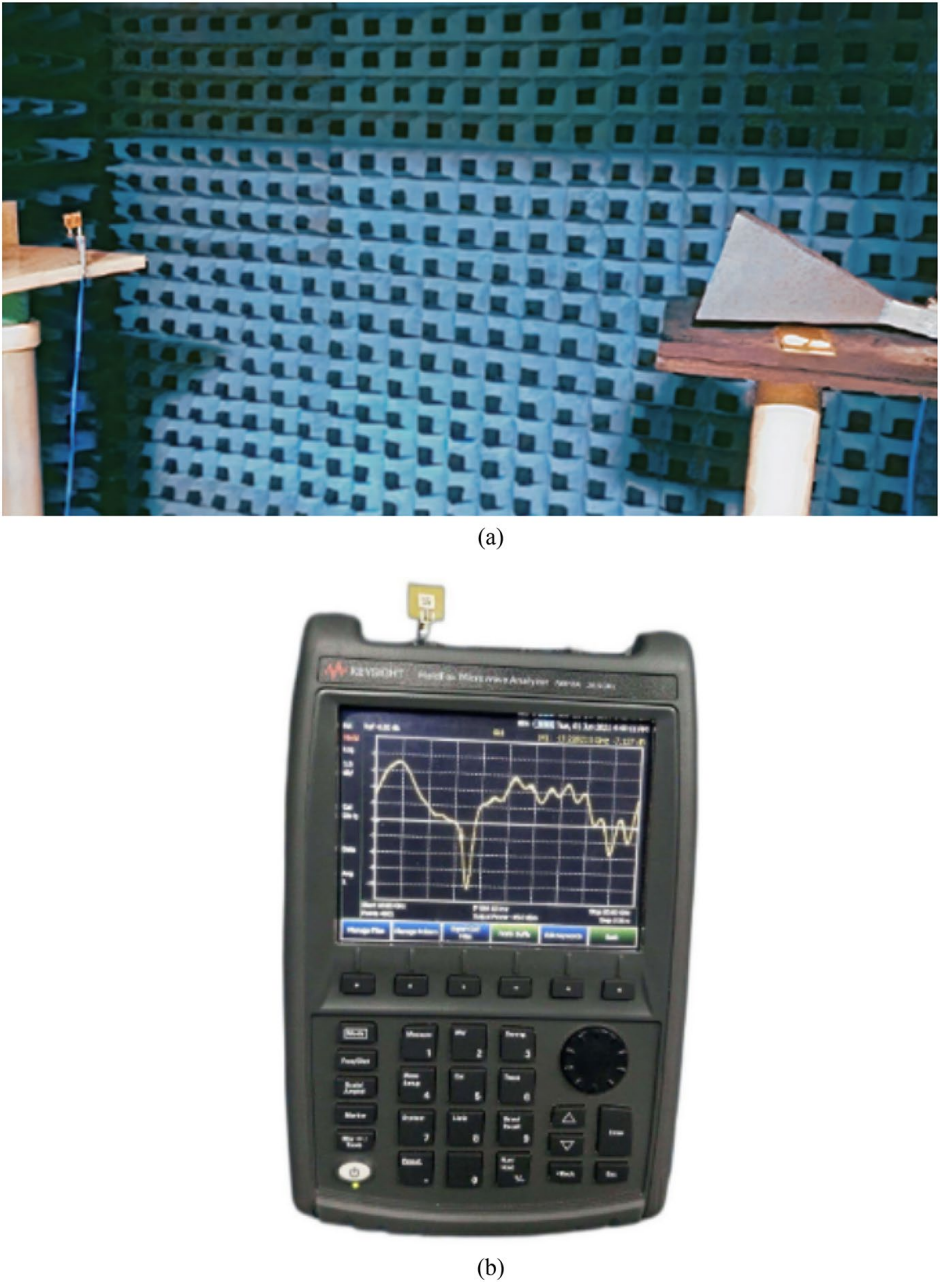
(**a,b**) Measured pattern setup for observing experimental results for the proposed antenna.

The gain of the proposed ECSRR metamaterial and EBG periodic structure, both with and without the ECCSRR bow-tie antenna, is exposed experimentally and numerically in Fig. 29. The measured result shows that the ECSRR metamaterial boosts gain by 5.2 dBi at 5.9 GHz. Embedded in the ECSRR EBG structure, the ECSRR bow-tie antenna’s envelope correlation coefficient is depicted in Fig. 30. At 5.57 GHz. The two-element MIMO antenna achieves its lowest ECC of 0.00081. Figure 31 shows the anechoic chamber chamber setup with proposed ECSRR bow-tie antenna with EBG structure. Table 7 shows a Comparative analysis of return loss, peak gain and performance of mutual coupling reduction of the Hybrid fractal antenna, 18 GHz and 28/38 GHz frequency reconfigurable antennas ECSRR bow-tie antenna with triangular ECSRR metamaterial Unit and ECSRR bow-tie antenna.

**Table 7:** Comparative analysis of return loss, peak gain and performance of mutual coupling reduction of the hybrid fractal antenna, 18 GHz and 28/38 GHz frequency reconfigurable antennas, ECSRR bow-tie antenna with triangular ECSRR metamaterial Unit and ECSRR bow-tie antenna.

Antenna	Antenna dimensions	Return loss (dB) (@resonating frequency, f_0_)	Peak gain (dBi) (@resonating frequency)	ECC	Band coverage isolation/mutual coupling reduction
Hybrid fractal antenna	8.1 × 5.5 × 1.6 mm	− 55.334 (@28 GHz), − 60.44 (@18.23 GHz), − 24.69 (@28.56 GHz), − 44.73 (@39.14 GHz), − 50.21 (@39.56 GHz), − 13.87 (@39.35 GHz)	4.41 (@18.23 GHz), 6.33 (@28 GHz), 7.70 (@39.5 GHz)	Nil	No mutual coupling reduction
18 GHz and 28/38 GHz frequency reconfigurable antennas	36 × 32 × 1.6 mm	− 10.176 (@27.26 GHz), (PIN diode Closed) − 9.95 (@29.2 GHz), (PIN diode Open) − 9.956 (@34.271 GHz), (PIN diode closed) − 10.034 (@42.19 GHz), (PIN diode Open) − 10.06 (@17.791 GHz), (PIN diode Closed) − 10.22 (@18.58 GHz), (PIN diode Open)	5.2 (@27.26 GHz) 4.9 (@29.2 GHz) 6.1 (@34.271 GHz) 5.7 (@42.19 GHz) 4.6 (@17.791 GHz) 5.7 (@18.58 GHz)	Nil	No mutual coupling reduction
ECSRR bow tie antenna	28 × 21 × 1.6 mm	− 12 (@2.5 GHz) − 28 (@3.1 GHz) − 45 (@5.58 GHz)	4.8 (@ 4.7 GHz), 2.9 (@ 4 GHz), 4.7 (@5 GHz), 4.35 (@5.5 GHz), 4 (@6 GHz)	Nil	No mutual coupling reduction
ECSRR bow-tie antenna with triangular ECSRR metamaterial Unit	28 × 49 mm (3 × 4 metamaterial)	− 22 (@3.3 GHz), − 13 (@4.2 GHz), − 12 dB (@4.8 GHz), − 11 (@5.2 GHz), − 18 (@5.8 GHz)	9.2 (@5.5 GHz), 5.14 (@5.9 GHz), 6 (@4 GHz), 7.3 (@5 GHz)	0.002 < 0.00235	< − 20 dB < − 15.1 dB − 20.2 dB

## Conclusion

This study comprehensively reviews the current developments in antenna array solutions for MIMO applications. In the first section of the manuscript, the difficulties of developing at 5G frequencies have been discussed; this has included a look at the constraints related to the channel propagation characteristics, the need for cost and energy-efficient system design, and the antenna integration aspects of miniaturization. The research provided a comprehensive overview of recent advancements in antenna technology for high-frequency applications, analyzing the pros and cons of the essential designs described in the scientific and technical literature. ECSRR bow-tie antennas simulated at 4.7 GHz yield 4.8 dBi maximum gain. The antenna measures 28 × 21 × 1.6 mm and achieves the maximum gains of 2.9 dBi, 4.7 dBi, 4.35 dBi, and 4 dBi for 4 GHz, 4.7 GHz, 5.5 GHz, and 6 GHz, respectively. The ECSRR EBG structure enhances gain in a CPW-fed microstrip quarter wave monopole antenna at 3.5–6.15 GHz. Mutual coupling reduction was investigated with the two-element ECSRR bow-tie antenna and ECSRR EBG structure. At 5.9 GHz and 9.2 dBi at peak, the bow-tie antenna’s ECSRR metamaterial periodic structure boosts gain. The mutual coupling and ECC were reduced in the 4–6 GHz frequency range thanks to the ECSRR EBG structure, which featured a two-element bow-tie antenna. It achieves 66 dB isolation at 4.9 GHz and − 32 dB mutual coupling across its working frequency range—the improvement in performance between the ECSR bow-tie antenna and the proposed ECSRR metamaterial with EBG periodic structure. At 5.9 GHz, the measured result demonstrates that the ECSRR metamaterial increases gain by 5.2 dBi. The envelope correlation coefficient of the ECSRR bow-tie antenna is incorporated into the ECSRR EBG structure. The two-element MIMO antenna has the best ECC performance at 5.57 GHz (0.00081). The ECSRR bow-tie antenna with triangular ECSRR metamaterial unit yields better mutual coupling reduction and undesired radiation. The proposed 6/18 GHz and 28/38 GHz frequency reconfigurable antenna provide a gain of 4.41 dBi, 6.33 dBi, and 7.70 dBi at 18 GHz, 28 GHz, and 38 GHz, respectively. 6G communication technologies will provide new options with wider frequency bands at higher frequencies.

## Data Availability

The datasets generated and/or analysed during the current study are not publicly available due to restrictions apply to the availability of these data, which were used under license for the current study, and so are not publicly available, but are available from the corresponding author on reasonable request.
